# Targeting the NRF2 pathway for disease modification in neurodegenerative diseases: mechanisms and therapeutic implications

**DOI:** 10.3389/fphar.2024.1437939

**Published:** 2024-07-25

**Authors:** Clara Mayer, Lluís Riera-Ponsati, Sakari Kauppinen, Henrik Klitgaard, Janine T. Erler, Stine N. Hansen

**Affiliations:** ^1^ NEUmiRNA Therapeutics, Copenhagen, Denmark; ^2^ Center for RNA Medicine, Aalborg University, Copenhagen, Denmark

**Keywords:** NRF2, BACH1, KEAP1, oxidative stress, neurodegeneration, neuroinflammation, mitochondrial dysfunction

## Abstract

Neurodegenerative diseases constitute a global health issue and a major economic burden. They significantly impair both cognitive and motor functions, and their prevalence is expected to rise due to ageing societies and continuous population growth. Conventional therapies provide symptomatic relief, nevertheless, disease-modifying treatments that reduce or halt neuron death and malfunction are still largely unavailable. Amongst the common hallmarks of neurodegenerative diseases are protein aggregation, oxidative stress, neuroinflammation and mitochondrial dysfunction. Transcription factor nuclear factor-erythroid 2-related factor 2 (NRF2) constitutes a central regulator of cellular defense mechanisms, including the regulation of antioxidant, anti-inflammatory and mitochondrial pathways, making it a highly attractive therapeutic target for disease modification in neurodegenerative disorders. Here, we describe the role of NRF2 in the common hallmarks of neurodegeneration, review the current pharmacological interventions and their challenges in activating the NRF2 pathway, and present alternative therapeutic approaches for disease modification.

## Introduction

Neurodegenerative diseases are a heterogenous group of neurological disorders characterized by the progressive loss of selective neuronal populations in the nervous system, giving rise to distinct clinical features, such as cognitive, behavioral, and motor symptoms (Agrawal, 2020). Amongst the most common neurodegenerative diseases are Alzheimer’s disease (AD), Parkinson’s disease (PD), Multiple Sclerosis (MS) and Amyotrophic Lateral Sclerosis (ALS). Importantly, the burden of neurodegenerative diseases on the healthcare system is projected to increase globally in the coming decades, due to the continuous population growth, an ageing society and the lack of early diagnostic biomarkers and effective medical treatments (Peplow et al., 2022). Despite their distinct clinical features and underlying pathophysiology, neurodegenerative diseases share fundamental disease processes associated with neuronal dysfunction and death (Erkkinen et al., 2018). Of those pathological characteristics, aberrant protein deposition, oxidative stress, neuroinflammation and mitochondrial dysfunction are believed to constitute the main molecular hallmarks (Wilson et al., 2023; Kovacs, 2019).

**Transcription factor nuclear factor-erythroid 2-related factor 2 (NRF2)** is a central regulator of the cellular defense line against toxic and oxidative insults. Its activation upregulates antioxidant defenses, inhibits inflammation, improves mitochondrial function, and maintains protein homeostasis (Cuadrado et al., 2019). As a member of the Cap ‘n’ Collar (CNC) transcription factor family, NRF2 exerts its function in the nucleus, by forming heterodimers with small musculoaponeurotic fibrosarcoma proteins (sMAFs), enabling NRF2 to bind to the enhancer region of the antioxidant response elements (AREs), which function as regulatory DNA sequences associated with the transcriptional activation of antioxidant enzymes and regulation of redox homeostasis (Katsuoka and Yamamoto, 2016).

**FIGURE 1:**
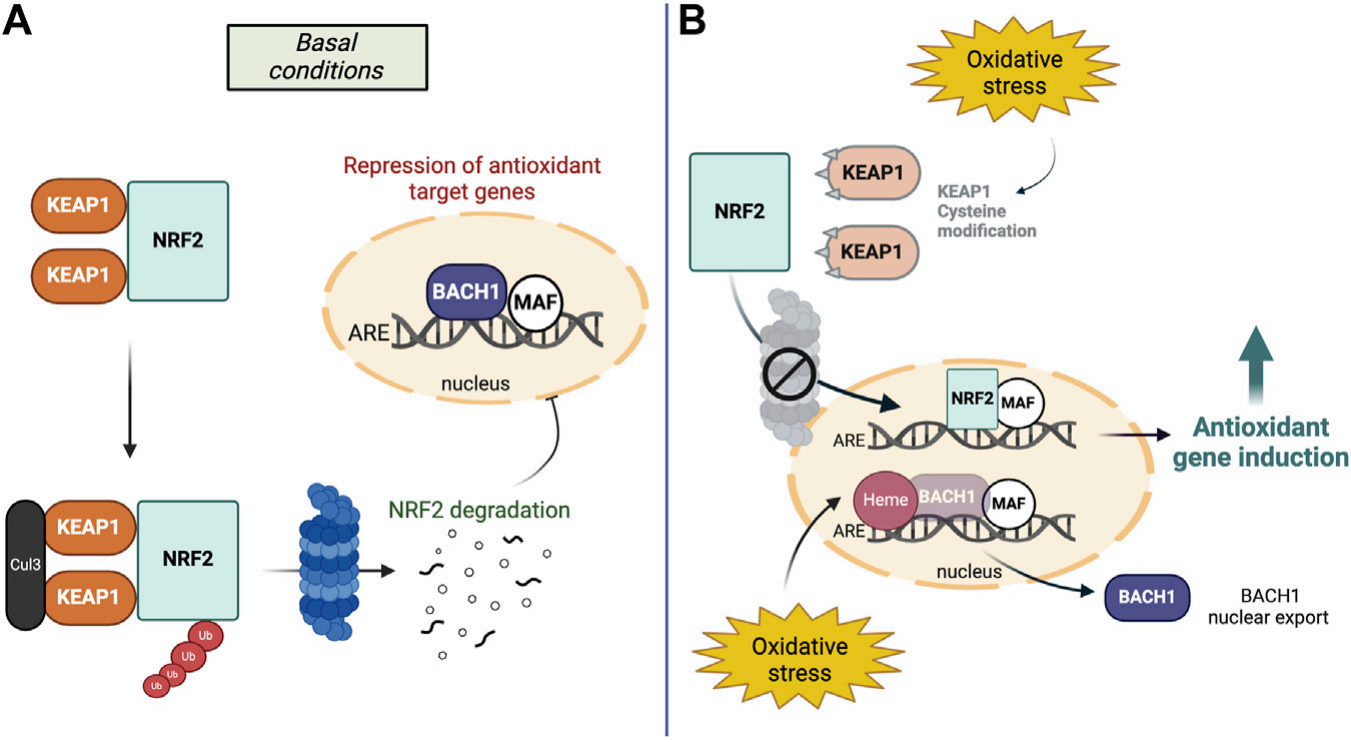
The NRF2-BACH1 pathway in basal conditions and oxidative stress. **(A)** Under basal conditions, two KEAP1 proteins bind to NRF2 and mediate constitutive ubiquitination and degradation of NRF2 in the cytosol. In the nucleus, BACH1-MAF homodimers inhibit the ARE binding site to inhibit the induction of antioxidant target genes. **(B)** During oxidative stress, KEAP1 proteins are modified at their reactive cysteine sites and NRF2 escapes its degradation. In the nucleus, oxidative stress increases levels of heme, BACH1s ligand and mediator for nuclear export. Therefore, NRF2 can replace BACH1 at the DNA binding site to activate its target genes.

NRF2 protein stability is tightly regulated and its two main regulators are i) Kelch-like ECH-associated protein 1 (KEAP1) in the cytoplasm and ii) BTB and CNC homology 1 (BACH1) in the nucleus. Under physiological conditions, KEAP1 mediates NRF2s constitutive proteasomal degradation (McMahon et al., 2003) while BACH1 acts as transcriptional antagonist of NRF2s target genes (Dhakshinamoorthy et al., 2005). During oxidative stress conditions, KEAP1 is inactivated by chemical modifications at its reactive cysteine residues, which allows NRF2 to escape degradation and for newly synthesized NRF2 to translocate to the nucleus (Zhang and Hannink, 2003). In turn, oxidative stress insults upregulate heme, BACH1s ligand and mediator for nuclear export, allowing for gene induction of NRF2-regulated cytoprotective factors (Ogawa et al., 2001; Reichard et al., 2007) (Figure 1).

In this article, we will review how NRF2 activity is associated with neurodegenerative diseases, particularly with disease hallmarks such as oxidative stress, neuroinflammation and mitochondrial dysfunction. We will then discuss the validity of the most common molecular targets; the cytoplasmic inhibitor KEAP1 and the transcriptional repressor BACH1, together with the different drug modalities serving as NRF2-activators. Finally, we will review the use of novel therapeutic approaches for activation of NRF2 as a disease-modifying treatment for neurodegenerative diseases.

## NRF2 coordinates cellular defense processes against diverse pathological hallmarks of neurodegeneration

Neurodegenerative diseases are characterized by common pathological hallmarks such as oxidative stress, neuroinflammation, mitochondrial dysfunction and protein aggregation. Targeting these features simultaneously holds the potential to achieve true disease modification, as it addresses the complexity and multifactorial nature of neurodegeneration. In accordance, targeting of NRF2 is of high interest, due to its prominent roles in several neurodegenerative hallmarks.

**Oxidative stress:** NRF2 induces antioxidant genes that reduce oxidative stress by minimizing the levels of reactive oxygen species (ROS) and reactive nitrogen species (RNS) (Ma, 2013). Those target genes include heme oxygenase 1 (HMOX-1), the main regulator of iron and heme metabolism (Suzen et al., 2022), NAD(P)H quinone dehydrogenase 1 (NQO-1), a reducer of ROS (quinones) (Ross and Siegel, 2021), and peroxiredoxin (PRX), which reduces peroxides (Chowdhury et al., 2009), among many others. Upregulation of these genes has been demonstrated to enhance neuronal resistance against oxidative insults (Satoh et al., 2006; Lim et al., 2008).

**Neuroinflammation:** NRF2 has been shown to be a central regulator of neuroinflammation (Saha et al., 2021). Mechanistically, it can dampen the pro-inflammatory response by decreasing the transcription of pro-inflammatory cytokines through direct DNA binding to Nuclear factor kappa-light-chain-enhancer of activated B cells (NF-κB) (Wang and He, 2022). In fact, these anti-inflammatory effects have been demonstrated in mouse microglia, macrophages, monocytes, and astrocytes (Kobayashi et al., 2016; Quinti et al., 2017).

**Mitochondrial dysfunction:** NRF2 also orchestrates mitochondrial proteins such as superoxide mutase 1 (SOD1), which scavenges mitochondrial ROS and RNS to protect the mitochondrial respiratory chain from oxidative insults (Cassina et al., 2008; Abdalkader et al., 2018), PTEN-induced putative kinase 1 (PINK1), a protein crucially important for sustaining functional mitochondrial homeostasis (Gumeni et al., 2021) and protein deglycase DJ-1 (PARK7), which maintains mitochondrial complex I activity and the mitochondrial membrane potential, two critical indicators for mitochondrial health (Hayashi et al., 2009; Im et al., 2012). Activation of NRF2-mediated antioxidant enzymes has been shown to enhance the biogenesis of mitochondria in mice and humans (Piantadosi et al., 2008; Merry et al., 2016; Hayashi et al., 2017) and by improving mitochondrial function, the excessive production of ROS was mitigated in mouse embryonic fibroblasts (Kovac et al., 2015).

**FIGURE 2:**
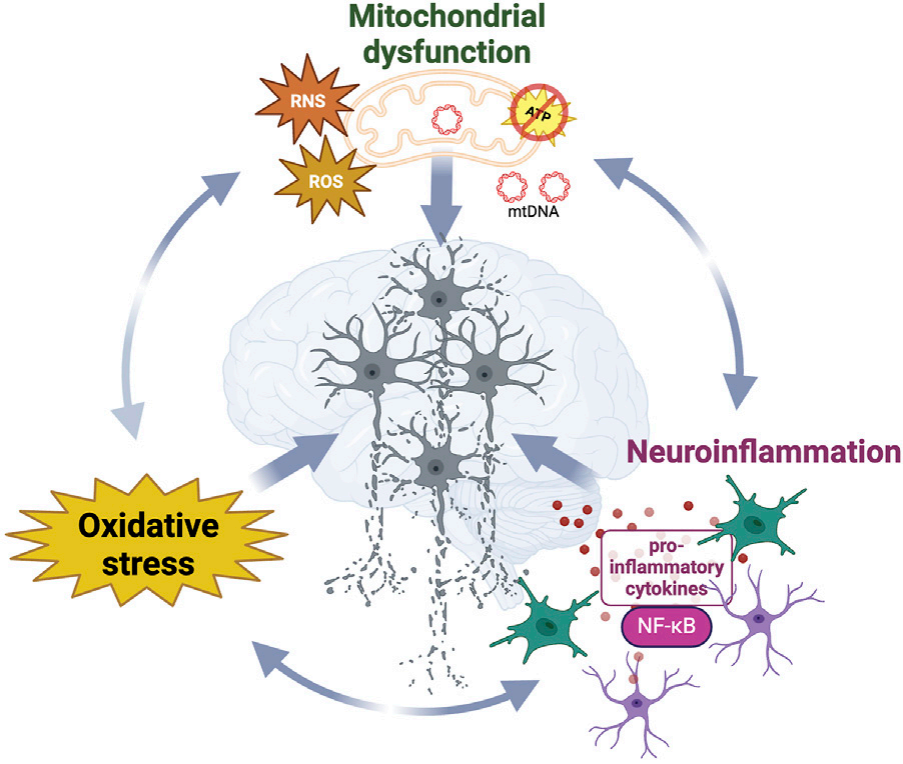
Synergistic interplay and dynamic relationships between neurodegenerative diseases hallmarks contribute to neurodegeneration. Mitochondrial dysfunction, oxidative stress and neuroinflammation reciprocally influence the progressive loss of structure and function in neurons in diseased brains. While damaged mitochondria significantly contribute to the increased generation of ROS and RNS, leading to oxidative stress which damages surrounding biomolecules, those reactive species and the release of mtDNA also act as danger signals activating the immune response.

Despite being considered as distinct pathological events, oxidative stress, neuroinflammation and mitochondrial dysfunction interplay with and enhance each other (Picca et al., 2020) (Figure 2). The main intracellular producer of ROS and RNS is the mitochondrial respiratory chain (Ray et al., 2012) and the increase of oxidative damage or release of mitochondrial DNA observed during mitochondrial dysfunction act as danger-associated signals (DAMPs), which initiate the immune response through pro-inflammatory NF-κB activation (Picca et al., 2020). While immune activation can effectively clear damaged mitochondria (Harding et al., 2023), immune dysfunction has been reported to trigger mitochondrial impairments and thereby increase the oxidative damage within the cell (Tresse et al., 2021). Taken together, the above findings illustrate the importance of the complex relationship between oxidative metabolism, inflammation, and neurodegeneration.

## Role of NRF2 in neurodegenerative diseases

Oxidative stress, neuroinflammation, and mitochondrial dysfunction are prevalent characteristics of the aging brain and research indicates that the expression of NRF2 and its downstream genes declines with age (Schmidlin et al., 2019). In fact, aging stands out as the main risk factor for the development and progression of neurodegenerative diseases (Hindle, 2010). Hence, NRF2 has emerged as an attractive target for the clinical intervention of neurodegeneration and in the following section, we describe the role of NRF2 in AD, PD, MS and ALS.

## NRF2 in Alzheimer’s disease

**AD** is the most common neurodegenerative disease and contributes to 60%–70% of the 55 million people diagnosed with dementia worldwide (WHO, 2023). Clinically, AD is characterized by progressive cognitive decline and pathologically, its two main hallmarks are intracellular neurofibrillary tangles comprised of the protein tau and extracellular β-amyloid (Aβ) plaques (Deture and Dickson, 2019). Emerging evidence suggests that Aβ plaques contribute synergistically to disease progression together with oxidative stress, inflammation and mitochondrial dysfunction, while this is less clear in regards to tau pathology (Brackhan et al., 2023). Studies have reported that Aβ plaques are tightly linked to oxidative stress (Allan Butterfield and Boyd-Kimball, 2018; Roy et al., 2023), since Aβ aggregation has been determined as source of oxidative stress (Karapetyan et al., 2022) and oxidative stress has been shown to increase the aggregation of Aβ (Mandal et al., 2022). Aβ plaques have also been associated with neuroinflammation (Leng and Edison, 2020; Sobue et al., 2023) and activated microglia has been reported to surround the protein depositions (Heneka et al., 2013), contributing to the spread of Aβ plaques (d’Errico et al., 2021) leading to an increase in pro-inflammatory cytokines (Rani et al., 2023). Additionally, Aβ has been observed to localize to mitochondria and to produce free radicals impairing the bioenergetic machinery (Manczak et al., 2004; 2006), while gene expression studies have identified a significant downregulation of genes related to the oxidative phosphorylation system in AD brains (Mastroeni et al., 2017).

In the AD brain, NRF2 has been mainly found within the cytoplasm and its nuclear fractions significantly reduced compared to age-matched healthy controls (Ramsey et al., 2007). Interestingly, the reduction of NRF2 levels has been associated with increased production of Aβ (Bahn et al., 2019). Consistent with these observations, molecular pathways that are known to be altered in AD brains, including severe amyloidopathy, tauopathy and exacerbated cognitive defects, were found dysfunctional in NRF2 knockout mice too (Rojo et al., 2017).

Conversely, NRF2 activation has been shown to exert beneficial effects in diverse disease models *in vitro* and *in vivo*. In AD astrocytes derived from pluripotent stem cells pharmacological activation of NRF2 ameliorated amyloid secretion and reduced inflammatory cytokine expression (Oksanen et al., 2020). In an AD mouse model (APP^NLGF^), increased expression of NRF2 reduced inflammation and oxidative stress and improved cognition (Uruno et al., 2020). Furthermore, several small molecules have been shown to activate the NRF2/ARE pathway and decrease Aβ pathology *in vitro* and *in vivo* (Ikram et al., 2019; Fakhri et al., 2020). In a clinical setting, therapies specifically targeting NRF2 have yet to be investigated, nevertheless, there is some evidence that treatment with antioxidant compounds could be beneficial. For instance, a clinical trial using Vitamin E as NRF2 activator revealed slowed decline in mini-mental state exam and activities of daily life (Dysken et al., 2014).

## NRF2 in Parkinson’s disease

**PD** constitutes the most common movement disorder, affecting over 8.5 million people worldwide (Ding et al., 2022). PD is characterized by the progressive loss of dopaminergic (DA) neurons in the substantia nigra pars compacta (SNpc) as well as the presence of α-synuclein-containing protein aggregates in the brain, referred to as Lewy bodies (Morris et al., 2024). Oxidative stress, dysfunctional mitochondria, and neuroinflammation have all been suggested to play roles in the onset and progression of PD (Navarro and Boveris, 2009; Di Filippo et al., 2010).

The presence of oxidative stress has been shown by of increased levels of oxidative damage to lipids, protein, and DNA in the parkinsonian brain (Alam et al., 1997; Mythri et al., 2011). These oxidative modifications can further activate microglia through DAMP-associated pathways and interestingly, α-synuclein misfolding has also been associated with microglia activation (Béraud and Maguire-Zeiss, 2012). Stimulation of the immune response has been demonstrated in various post-mortem studies, which found elevated levels of pro-inflammatory cytokines (Nagatsu et al., 2000; Brodacki et al., 2008; Reale et al., 2009) and additional evidence suggests that several proteins, which are highly expressed by microglia and astrocytes, such as Leucine-rich repeat kinase 2 (LRRK2), PINK1 and PARKIN, are encoded by genes implicated in familial forms of PD (Scarffe et al., 2014; Brockmann et al., 2016; Isik et al., 2023) Furthermore, excessive production of ROS has also been linked to dysfunctions in mitochondrial complex I and the complex’ activity reported to be reduced in PD patient brains (Keeney et al., 2006). Coherently, complex I inhibitors like the neurotoxin 1-methyl-4-phenyl-l,2,3,6-tetrahydropyridine (MPTP) lead to PD-like symptoms in humans and animals, accompanied by the loss of adenosine triphosphate (ATP) production and increased generation of oxidative stress (Smith and Bennett, 1997). Additionally, mtDNA damage has been associated with PD etiology and progression (Tresse et al., 2023) and mitochondrial dysfunction is linked genetically to PD, since many mutations associated to familial cases of PD were found in genes such as PINK1 or PARKIN, which regulate mitochondrial turnover (Valente et al., 2004; Lev et al., 2006; 2008; Paterna et al., 2007; Devi et al., 2008).

Failure of the NRF2 pathway has been reported in PD. Pathway dysregulation has been observed in the brain, urine and plasma of PD patients, showing a significant reduction of antioxidant enzyme activity along with an increased level of oxidative stress (Colamartino et al., 2018). Also, mouse models of PD have indicated decreased NRF2 activity in astrocytes (Chen et al., 2009) and knockdown of NRF2 has been shown to cause an increase in mitochondrial ROS production (Kovac et al., 2015), the loss of dopaminergic neurons in association with microglia activation (Rojo et al., 2010) and exacerbation of synuclein aggregation (Lastres-Becker et al., 2012).

Importantly, NRF2 activation has been shown to alleviate PD-associated pathological hallmarks, specifically oxidative stress, mitochondrial dysfunction and neuroinflammation through expression of its target genes (Yang et al., 2022). In the MPTP mouse model, NRF2 activation has been associated with a reduction in oxidative stress (Williamson et al., 2012), induction of antioxidant enzymes (Jazwa et al., 2011), and improvement of the mitochondrial respiratory rate (Fu et al., 2018). Anti-inflammatory properties of NRF2 signaling have been verified in LPS-stimulated mouse microglia, where its upregulation reduced inflammatory markers (Townsend and Johnson, 2016) and astrocytic, and microglial activation were found to be significantly lower (Jing et al., 2015). Additionally, treatment with NRF2 activators in PINK1- or PARKIN-deficient *drosophila* restored mitochondrial function, elevated mitophagy rates and suppressed oxidative stress (Gumeni et al., 2021). In conclusion, these data unveil the therapeutic potential of increasing NRF2 signaling for treating PD.

## NRF2 in Multiple Sclerosis

**MS** is a chronic neurodegenerative and autoimmune disease that affects 2.8 million people worldwide and remains one of the most common causes of neurological disability in the young adult population (Jakimovski et al., 2024). Its main pathological hallmark constitutes demyelinating lesions that accumulate within the central nervous system (CNS) (Popescu et al., 2013; Filippi et al., 2018) leading to cognitive and motor disability. The demyelinating process is accompanied, amongst other factors, by extensive neuroinflammation (Gold et al., 2006). Elevated levels of proinflammatory cytokines (Khaibullin et al., 2017) and increased levels of chemokines responsible for recruiting peripheral immune cells and activating the adaptive immune response (Kalinowska-Łyszczarz et al., 2011), have been found in MS patients. Additionally, altered expression of genes involved in the activation of the adaptive immune response have been observed and provide the genetic link between MS and progressive neuroinflammation (Ramanathan et al., 2001). The prolonged activation of macrophages and microglia has been associated with the increased production of ROS (Hemmati et al., 2020) and eventually results in oxidative stress (Zhang et al., 2020). This has been demonstrated in MS patients as increased concentrations of ROS (Acar et al., 2003; Gilgun-Sherki et al., 2004) along with decreased antioxidant levels (Choi et al., 2018; De Riccardis et al., 2018) were found in serum and cerebrospinal fluid. The abundant generation of ROS along with the enhanced neuroinflammatory response then negatively affect mitochondrial function (Forte et al., 2007; Blagov et al., 2022). As observed in human studies, where mitochondrial oxidative phosphorylation complexes were decreased in the MS cortex (Witte et al., 2013) together with mutated mtDNA and reduced mtDNA copy numbers (Ban et al., 2008; Blokhin et al., 2008).

The role of NRF2 on MS pathogenesis becomes observable in both disease models as well as patients. The experimental autoimmune encephalomyelitis (EAE) or lipopolysaccharide (LPS)-induced MS effectively recapitulate the inflammatory response, mitochondrial dysfunction, and oxidative stress (Wang et al., 2017; Yang et al., 2018). In those, loss of NRF2 resulted in a more rapid onset and more severe clinical course of the EAE model, which was accompanied by increased glial activation, exacerbated spinal cord damage, and axonal degeneration as well as increased levels of proinflammatory cytokines (Johnson et al., 2010; Larabee et al., 2016). Additionally, NRF2-activators have shown beneficial effects in MS model systems. These effects include the reduction of LPS-induced neurotoxicity, lowering inflammatory markers and gliosis, and improvement of synaptic and mitochondrial function (Larabee et al., 2016; Chen et al., 2017; Khan et al., 2018; Rehman et al., 2019).

In fact, NRF2 activation as a clinical target is well established for the treatment of MS since the potent NRF2-activator dimethyl fumarate (DMF) was approved by FDA as first disease-modifying agent in 2013 (Fox et al., 2012; Gold et al., 2012). DMF has been shown to alleviate hallmarks of neurodegenerative diseases through the blockage of pro-inflammatory NF-κB (Gillard et al., 2015), the upregulation of the antioxidant NRF2-ARE pathway (Rosito et al., 2020), and by increasing mitochondrial function (Hui et al., 2021). Importantly, MS patients who showed a significant increase in the expression of NRF2’s downstream target, NQO-1, following DMF treatment were more likely to achieve no evidence of disease activity after 1 year (Hammer et al., 2018).

## NRF2 in Amyotrophic Lateral Sclerosis

**ALS** is the most common motor neuron disease in adults and estimates have reported that 31,000 patients live with ALS in the US, where on average 5,000 new patients are diagnosed annually (Mehta et al., 2023). ALS is characterized by neurodegeneration of motor neurons in brain, spinal cord, and periphery, leading to progressive muscle paralysis: terminal events often comprise aspiration pneumonia and respiratory insufficiency (Morgan and Orrell, 2016; Taylor et al., 2016). Approximately 90%–95% of ALS cases appear sporadically, whereas ca. 5%–10% show a familial predisposition (Andersen and Al-Chalabi, 2011). The most frequent genetic mutations and defects are found in SOD1 (Ghasemi and Brown, 2018), a gene encoding an essential copper/zinc antioxidant enzyme which is likely involved in both familiar and sporadic presentations (Tafuri et al., 2015).

Notably, mutations and defects in SOD1 have been associated with oxidative stress, neuroinflammation and mitochondrial dysfunction. Increased oxidative damage has been detected in both sporadic and familial ALS patients carrying SOD1 mutations (Abe et al., 1995; Beal et al., 1997; Blasco et al., 2017). Post-mortem studies have described increased activation of microglia and astrogliosis (Chiot et al., 2020) and elevated levels of inflammatory cytokines have been reported in ALS patient’s serum (Tortelli et al., 2020). Being a mitochondrial antioxidant enzyme, mutated SOD1 has further been observed to aggregate at the outer mitochondrial membrane and is thought to lead to mitochondrial damage including morphological changes, excessive superoxide production, increase in mitochondria membrane permeability and defective mitophagy (Higgins et al., 2003; Tak et al., 2020).

Importantly, mutant SOD1 has been demonstrated to decrease the expression of antioxidant transcription factor NRF2 and its dependent target genes in an established NSC134 cellular model for SOD1-associated ALS (Kirby et al., 2005). In post-mortem patient samples, both messenger RNA (mRNA) and protein levels of NRF2 have been reported to be reduced (Sarlette et al., 2008) and proteomic analysis of spinal motor neurons from humans with SOD1-related ALS had revealed that the NRF2-induced gene peroxiredoxin is transcriptionally repressed (Wood-Allum et al., 2006). It is important to note that a recent study has demonstrated a significant upregulation of NRF2, HMOX-1 and NQO-1 mRNA expression, as well as an increase in HMOX-1 and NQO-1 protein levels in the spinal cord of ALS patients (Lastres-Becker et al., 2022). However, their results have also demonstrated increased oxidation of lipid substrates, indicating elevated oxidative stress levels (Lastres-Becker et al., 2022), which suggests that the NRF2-mediated antioxidant machinery may be activated but to an insufficient degree in ALS.

In support of the NRF2 pathway being a potential target for the treatment of ALS, studies have shown that NRF2 overexpression in astrocytes provided neuroprotection against mutated SOD1-induced toxicity and increased the median survival of mutant SOD1 transgenic mice (Vargas et al., 2008; 2013). *In vitro* studies have demonstrated that overexpression of Angiogenin, a RNase associated with familial ALS, activates NRF2 which protects astrocytes from oxidative stress (Hoang et al., 2019) and curcumin mediated NRF2 induction ameliorates mitochondrial dysfunction (Lu et al., 2012). Further evidence of NRF2s beneficial effects on ALS pathology were reported by clinical studies, which have shown that pharmacological activation of NRF2 improved the disease progression of ALS patients and reduced oxidative stress (Ahmadi et al., 2018; Chico et al., 2018).

**FIGURE 3:**
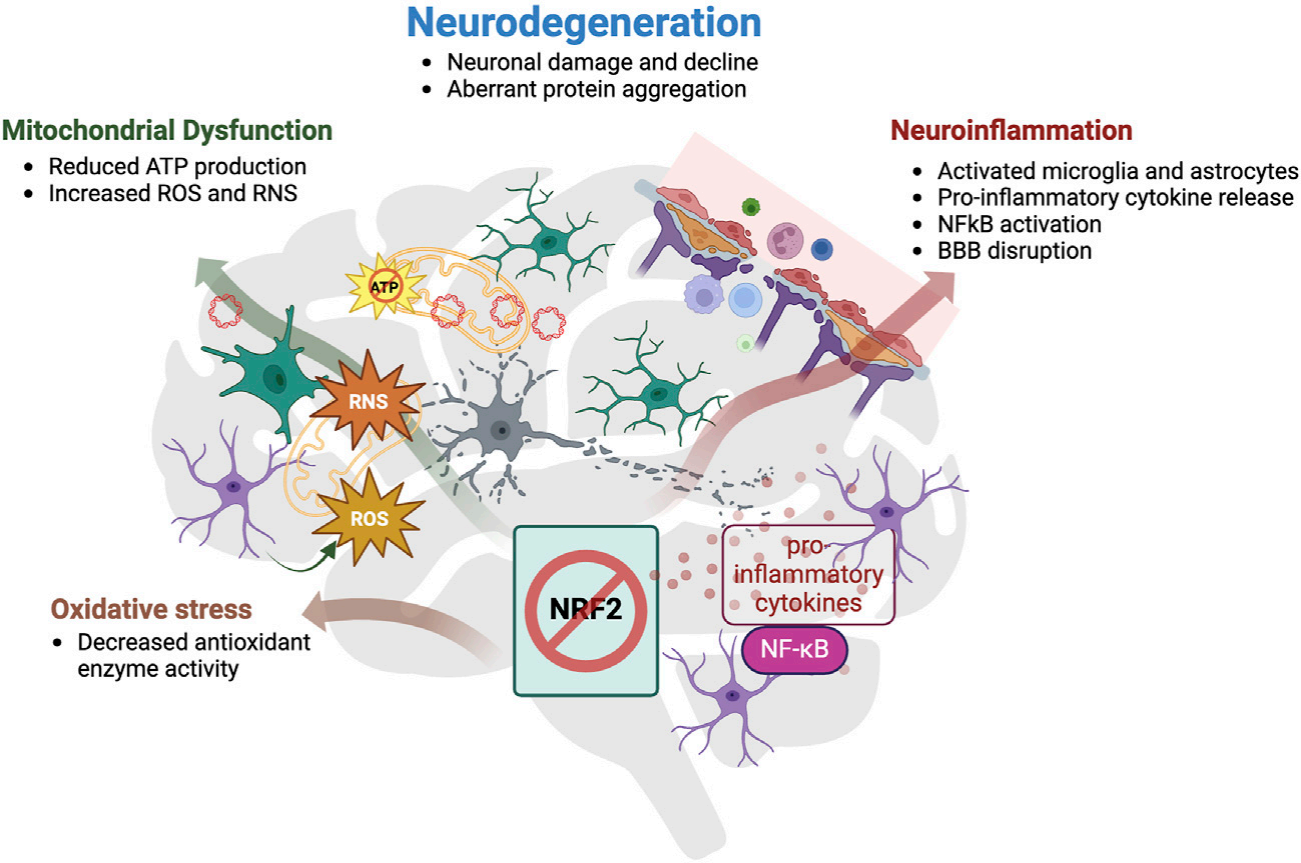
Summary of the effects of reduced activity or loss of NRF2 in the brain. Loss or reduced activity of NRF2 enhances oxidative stress, mitochondrial dysfunction and neuroinflammation, which altogether contribute to neurodegeneration. Impaired NRF2 signaling was associated with a decrease in antioxidant enzyme activity, which is responsible for the incapability of cells to remove oxidative insults and therefore results in higher exposure to oxidative stress. Mitochondria are both a major source, and immediate target of oxidative stress. Reduced mitochondrial membrane potential hinders the production of chemical energy ATP, and simultaneously, increases the generation of ROS and RNS, rendering the enzymes responsible for eliminating those reactive species inefficient. Discarded mtDNA can then act as DAMP and trigger danger-signal receptors in microglia, which will further activate astrocytes and together, they induce a broad pro-inflammatory cytokine response through transcription factor NF-κB. Prolonged immune activation disrupts the blood brain barrier (BBB) permeability and allows more immune cells to infiltrate and intensify the immune reaction. Cooperatively, these three pathological hallmarks count, amongst others, as causes of neurodegeneration, which comprises neuronal damage and decline, including dysfunctional neuronal signaling, and aberrant protein aggregation.

Collectively, these data show that the NRF2 pathway is dysfunctional in various neurodegenerative diseases and the effects of the reduced or lost activity of the NRF2-mediated antioxidant pathway in the brain are summarized in Figure 3. Furthermore, those findings suggest that the NRF2 pathway plays an important role in protecting neuronal function as well as in preventing cognitive decline through regulation of a multitude of pathological pathways implicated in neurodegenerative diseases. Thus, targeting the NRF2 pathway holds great potential for being a disease-modifying treatment.

## Targeting the NRF2 pathway for disease modification in neurodegenerative diseases

To date, conventional treatment options for neurodegenerative diseases only provide symptomatic relief, and do not interfere with the underlying neuropathological processes. The majority of clinical trials investigating disease-modifying therapies have sought to halt or reverse the deposition of specific proteins in the brain and, while the first disease-modifying drug targeting protein deposition has been approved for AD (Cummings, 2023), two different phase II clinical trials of antibodies targeting α-synuclein in PD patients have not shown any effect on symptom relief (Kharel and Ojha, 2023). Unfavorably, when these protein inclusions are diagnosed, clinical manifestations have started to appear, and underlying pathological hallmarks are greatly advanced (Kalia and Lang, 2015; Ower et al., 2018), which hinders early and successful therapeutic intervention. Furthermore, neurodegenerative diseases develop from a multitude of pathological hallmarks, hence, the therapeutic targeting of only one of them might not have a significant effect on the course of a heterogenous disorder.

Developing effective drugs that can modify the progression of neurodegenerative diseases is crucial and would represent a major medical breakthrough. Due to their complexity, unclarified pathogenesis, progressive and severe nature, it is likely that a truly disease-modifying therapy for neurodegenerative diseases needs to regulate several pathological pathways simultaneously (Li et al., 2020). Hence, pharmaceuticals that target the NRF2 pathway can influence multiple pathways involved in the neurodegenerative process, and therefore hold potential to become therapies that can significantly modify the course of neurodegenerative diseases.

Numerous compounds that activate NRF2 through various mechanisms and structural compositions have been identified (Amoroso et al., 2023). NRF2 activators include naturally occurring molecules such as phytocompounds and cannabinoids, semisynthetic molecules such as Omaveloxolone, or dimethyl fumarate (DMF), the longest NRF2 activator in clinical use (Bomprezzi, 2015). Herein, we discuss the credibility and efficacy of the most prevalent class of NRF2 activators, namely, KEAP1 inhibitors, while also introducing a novel focal point within the NRF2 pathway: the transcription factor BACH1.

## Pharmacological activation of NRF2: KEAP1-inhibitors

Thus far, the most common class of NRF2-activators mimic the inhibition of NRF2s cytoplasmic negative regulator KEAP1, an adaptor protein for the CUL3/RBX1 E3 ubiquitin ligase complex, to prevent NRF2 proteasomal degradation (Kobayashi et al., 2004). With regards to neurodegenerative diseases, the two following described compounds, DMF and Sulforaphane (SFN), are amongst the most well-studied compounds of the KEAP1-inhibitory category. Nevertheless, it is worth mentioning that other compounds, such as Curcumin (Shahcheraghi et al., 2022), Resveratrol (Yadav et al., 2022) or Ursodiol (Huang, 2021) also have been reported to exert activating effects on NRF2 in neurodegenerative diseases.

**Dimethyl Fumarate** (DMF, Tecifidera^®^) is an NRF2 activator in clinical use for the treatment of relapsing forms of MS and psoriasis. DMF has been shown to alleviate hallmarks of neurodegenerative diseases such as inflammation and oxidative stress through the blockage of pro-inflammatory NFkB (Gillard et al., 2015) and upregulation of the antioxidant NRF2-ARE pathway (Rosito et al., 2020). This drug has been demonstrated to exhibit several distinct modes of actions, one of them being the irreversible modulation of KEAP1 active cysteine residues (Piroli et al., 2019). Furthermore, studies have shown that DMF causes BACH1 nuclear exit in rat dopaminergic neurons and human cells (Ahuja et al., 2022) (Table 1). These observations indicate that DMFs activation of NRF2 originates not only from inducing NRF2 in the cytoplasm but also from inhibiting the NRF2 repressor in the nucleus. In SH-SY5Y cells stimulated with Aβ_1–42_ or 6-OHDA, DMF has been reported to increase cellular viability and reduce intracellular ROS production, both through the activation of HMOX-1, while a study treating ALS patient-derived macrophages demonstrated DMFs ability to inhibit pro-inflammatory cytokines as well as NF-kB (Table 1). Furthermore, AD and PD studies in mice found higher levels of antioxidant enzymes and a reduction of inflammatory markers in the brain, as well as improvement of behavioral impairment upon treatment with DMF (Table 1). Collectively, these data show that DMF constitutes an attractive compound for the pharmaceutical industry for drug repurposing in other neurodegenerative diseases (Bresciani et al., 2023).

**TABLE 1:** DMF’s ability to alleviate pathological hallmarks of AD, PD and ALS in pre-clinical models in the context of Nrf2 activation. This table summarizes the impact of DMF on key pathological features observed in pre-clinical models of AD, PD, and ALS. The data highlights DMF’s ability to modulate disease hallmarks through the activation of the NRF2 pathway. Additionally, the table details the extent of neuroprotection, oxidative stress reduction, and anti-inflammatory effects achieved by DMF treatment in the various disease models.

Disease	Experimental model	Treatment regimen	Major outcomes	References
Alzheimer’s Disease *in vitro*	SH-SY5Y cells; stimulated with Aβ_1-42_	DMF (30 μM)	Increase in cell viability; actvation of HMOX-1	Campolo et al. (2018)
Alzheimer’s Disease *in vivo*	C57/BL6J male mice; intrahippocampal injection of Aβ_1-42_ and IBO	DMF (48 mg/kg)	Rescue of Aβ-induced memory impairment; delayed hippocampal atrophy, upregulation of antioxidant enzymes via NRF2; inhibition of lipid peroxidation, apoptosis, inflammation, mitochondrial dysfunction, and reduction in Aβ deposition	(Sun et al., 2022)
	57/BL6j-NRF2-WT and C57/BL6j-NRF2-KO mice	DMF (100 mg/kg)	NRF2-deficiency accelerates mouse death; treatment with DMF shows reduction in inflammatory markers and improvement of motor and memory cues	Rojo et al. (2018)
Parkinson’s Disease *in vitro*	SH-SY5Y cells; 6-OHDA-induced damage	DMF (1–4 μM)	Activation of Nrf2 and downstream genes; reduction of intracellular ROS production and cell death signals	Jing et al. (2015)
	Rat N27 dopaminergic and human M17 neuroblastoma cells	DMF (10 or 20 μM)	DMF activates Nrf2 via S-alkylation and Bach1 nuclear export; upregulation of HMOX-1 mediates neuroprotection	Ahuja et al. (2016)
Parkinson’s Disease *in vivo*	C57BL/6 mice; 6-OHDA lesions	DMF (50 mg/kg)	Activation of Nrf2-mediated ARE-gene transcription in striatum; reduction of oxidative stress and inflammation markers; elevation in striatal dopamine and its metabolites	Jing et al. (2015)
	Male CD1 mice; MPTP-induced PD	DMF (10, 30, and 100 mg/kg)	Reduction of behavioral impairments and degeneration of dopaminergic tract; increase in NRF2 antioxidant target genes	Campolo et al. (2017)
	Mice; human α-syn. Gene rAAV6-delivery to SNpc	DMF (100 mg/kg)	Decrease of microgliosis and astrogliosis; higher levels of antioxidative markers	Lastres-Becker et al. (2016)
Amyotrophic Lateral Sclerosis *in vitro*	Differentiated macrophages from ALS patients	DMF (0.1 μM)	Inhibition of pro-inflammatory cytokines, and NF-kB targets	Zamiri et al. (2023)

**Sulforaphane** was the first identified and most potent naturally occurring NRF2 activator found in cruciferous vegetables such as broccoli, cauliflower, and Brussel sprouts (Zhang et al., 1992; Yagishita et al., 2019). SFN has been shown to exert antioxidant, anti-inflammatory and antiapoptotic effects through suppression of NRF2’s physiological inhibitors KEAP1 and Glycogen synthase kinase-3 beta (GSK-3β) (Zheng et al., 2022). In combination with its excellent bioavailability in the central nervous system, these properties have led to the compound gaining interest from the scientific community for the treatment of neurodegenerative diseases. *In vitro* models of AD, PD and MS have demonstrated that SFN is efficient in counteracting cell death, neuroinflammation, oxidative stress and protein-aggregation induced by various toxins such as A𝛽 plaques, LPS, 6-OHDA or hydrogen peroxide, through upregulation of the NRF2/ARE-axis (Table 2). Moreover, the phytocompound has even been reported to improve cognitive and motor deficits in mouse studies of the same diseases (Schepici et al., 2020) (Table 2).

**TABLE 2:** Sulforaphane provides neuroprotection in NDD models through activation of the antioxidant NRF2/ARE pathway This table illustrates the neuroprotective properties of SFN in various pre-clinical models of AD, PD and MS. Key outcomes include the reduction of oxidative stress, mitigation of neuronal damage, and enhancement of cellular defense mechanisms. The table also highlights the improvements in cognitive and motor functions observed *in vivo* disease models following SFN treatment.

Disease	Experimental model	Treatment regimen	Major outcomes	References
Alzheimer’s Disease *in vitro*	SH-SY5Y cells and APP + PS transgenic 5xFAD and 3xTg-AD mice	SFN (1 μM)	Upregulation of Nrf2 reduced amyloidogenesis and activation of its antioxidant target genes HMOX-1 and NQO-1 alleviated oxidative stress	Bahn et al. (2019)
	SH-SY5Y cells; A*β*_25–35_-induced oxidative cell death	SFN (1,2 and 5 μM)	Activation of Nrf2, HMOX-1 and NQO-1 reduced Aβ-induced cell death and oxidative stress	Lee et al. (2013)
	SH-SY5Y cells; A*β*_25–35_-induced oxidative cell death	Crude juices of broccoli sprouts (10 μM)	Upregulated cellular antioxidant defense capacity by increased HMOX-1 mRNA levels and NQO-1 activity; activation of nuclear translocation of transcription factor Nrf2 moderating antioxidant gene expression	Masci et al. (2015)
	BV2 murine microglia; activated by LPS	SFN-enriched broccoli sprouts 10 MI	Increased expression of Nrf2-HO1 axis reduced inflammation, oxidative stress, and apoptosis	Subedi et al. (2019)
	Neuroblastoma N2a/APPswe cells	SFN (1.25 and 2.5 μM)	Upregulation of Nrf2 expression, promoted its nuclear translocation and increased mRNA levels of HMOX-1 and NQO-1 inhibited oxidative stress and reduced neuroinflammation	Zhao et al. (2018)
	Human THP-1 macrophages; induced by Aβ_1-40_	SFN (5 μM)	Reduction of neuroinflammation and inhibition of oxidative stress via Nrf2 and target gene upregulation	An et al. (2016)
Alzheimer’s Disease *in vivo*	Sprague-Dawley rats, administered Aβ-oligomers	SFN (5 mg/kg)	Improved depressive behaviors, reduced oxidative stress and neuroinflammation	Wang et al. (2020)
	C57BL/6 mice AD-lesions; induced by administration of D-galactose and aluminium	SFN (25 mg/kg)	Improved cognitive and motor deficits, reduced oxidative stress, decrease in formation of Aβ-plagues	Zhang et al. (2015)
	ICR mice; Aβ-induced AD	SFN (30 mg/kg)	Improved cognitive deficits, prevention of Aβ-aggregation, reduced oxidative stress and neuroinflammation	Kim et al. (2013)
	3×Tg-AD mice	SFN (10 mg/kg)	Improvement in cognitive deficits, reduced oxidative stress and Aβ-aggregation	Lee et al. (2018)
Parkinson’s Disease *in vitro*	SH-SY5Y cells and mouse embryonic fibroblasts	SFN (5 μM)	Reduced oxidative stress and cell death dependent on the Nrf2-Keap1 pathway	Niso-Santano et al. (2010)
	PC-12 cells; induced by MPP+	SFN (0.5, 1.0, 2.5, 5.0 and 10 μmol/L)	SFN restored Nrf2, HMOX-1 and NQO-1 levels upon MPP + induced cytotoxicity and thereby prevented oxidative damage Reduction of oxidative stress (6-OHDA) -induced cell damage and increase in cell viability	Bao et al. (2019)
	PC-12 cells; 6-OHDA -induced cell damage	SFN (0.1, 1 and 5 μM)	SFN showed cytoprotective effects by inhibiting 6-OHDA-induced ER stress via activation of Nrf2	Deng et al. (2012a)
	PC-12 cells; 6-OHDA -induced cell damage	SFN (1 and 5 μM)	SFN induced the translocation of Nrf2 into the nucleus and increased HMOX-1 expression	Deng et al. (2012b)
	Dopaminergic neurons of organotypic rat nigrostriatal cultures; 6-OHDA induced neuronal death	SFN (5 μM)	Reduction of nigrostriatal neurodegeneration	Siebert et al. (2009)
	Mouse primary cortical neurons; induced by 5-S-cysteinyl-dopamine- toxicity	SFN (0.01 and 1 μM)	Protection from cell death and inhibition of oxidative stress via activation of the Nrf2 pathway	Vauzour et al. (2010)
	CATH.a and SK-N-BE 2) C and mesencephalic dopaminergic neurons induced by 6-OHDA 6and tetrahydrobiopterin	SFN (0.5, 1, 2.5 and 5 μM)	Reduction of oxidative stress and increase of NQO-1 activity	Ji et al. (2007)
	SH-SY5Y cells; cell toxicity; induced by 6-OHDA	SFN (0.63–5 μM/L)	Prevention of oxidative stress and neuronal damage; increase in NQO-1 activity	Tarozzi et al. (2009)
	SH-SY5Y cells; oxidative treatment with 6-OHDA and C57Bl/6 6-OHDA-PD mice	SFN (0.1–5 μM) SFN (30 μmol/kg)	Higher active nuclear Nrf2 protein and increased mRNA Nrf2 levels explain neuroprotective effects of SFN; confirmed in mouse model	Morroni et al. (2018)
Parkinson’s Disease *in vivo*	C57Bl/6 mice; rotenone-induced neurotoxicity	SFN (50 mg/kg) I	Prevention of motor deficits and loss of dopaminergic neurons; increased expression of Nrf2, HMOX-1 and NQO-1 reduced oxidative stress	Zhou et al. (2016)
	C57Bl/6; 6-OHDA-PD mice	SFN (5 μM)	Improvement of motor-deficits, protection from neurodegeneration and neuronal apoptosis, reduced oxidative stress	Morroni et al. (2013)
	Wild-type and Nrf2-KO mice in the MPTP-PD model	SFN (50 mg/kg)	In wild-type mice, attenuation of nigrostriatal neurodegeneration and neuroinflammation by upregulation of Nrf2	Jazwa et al. (2011)
Multiple Sclerosis *in vitro*	OLN-93 cells; induced by hydrogen peroxide	SFN (5 μM)	Prevention of oxidative stress through increased activation of antioxidant enzymes	Lim et al. (2016)
	Primary co-cultures of rat astroglial and microglial cells stimulated by LPS	SFN (1, 5 or 15 μM)	Reduction of inflammatory mediators and enhancement of detoxification mechanisms	Wierinckx et al. (2005)
Multiple Sclerosis *in vivo*	C57Bl/6 mice in the EAE model	SFN (50 mg/kg)	Reduction of oxidative stress via activation of the Nrf2 pathway, inhibition of inflammation, improvement of behavioral deficits	Li et al. (2013)

## Pharmacological activation of NRF2: BACH1 inhibitors

BACH1 antagonizes NRF2 at the DNA binding site of antioxidant genes and thereby represses the expression of NRF2s target genes. In fact, nuclear exclusion of BACH1 has been reported to be necessary for the activation of neuroprotective pathways through its antioxidant target gene: HMOX-1, the rate-limiting enzyme in heme catabolism (Reichard et al., 2007). Despite the therapeutic potential of the NRF2-BACH1 axis, very few BACH1 inhibitors, namely, heme, cannabidiol (CBD) and HPPE (N-(2-(2-hydroxyethoxy)ethyl)-1-methyl-2-((6-(trifluoromethyl)benzo[d]thiazol-2-yl)amino)-1H-benzo[d]imidazole-5-carboxamide have been tested in pre-clinical settings of neurodegenerative diseases up to date.

Heme is BACH1s own endogenous ligand and its oxidized form, hemin, is the primary compound used to inhibit BACH1. An AD *in vitro* study demonstrated heme-mediated increase of the basal expression of NRF2 antioxidant target genes (Zhang et al., 2021) (Table 3). However, the development of heme as a safe therapeutic agent presents significant challenges, as it catalyzes the formation of ROS upon interaction with atmospheric oxygen, potentially leading to cellular damage and oxidative stress. Another compound that was identified as potent BACH1-inhibitor was the phytocannabinoid cannabidiol (CBD), which induces nuclear export and degradation of the transcription factor (Casares et al., 2020). *In vitro* models have confirmed CBDs antioxidant and anti-inflammatory properties, acting through HMOX-1 and inducing the downregulation of pro-inflammatory cytokines (Juknat et al., 2016; Duvigneau et al., 2020). In an AD mouse model, CBD treatment reduced neuroinflammation and facilitated neurogenesis in the hippocampus, although this was thought to be mainly mediated through PPARγ (Esposito et al., 2011) (Table 3). Recently, a study identified HPPE, a substituted benzimidazole, as BACH1 inhibitor coupled to activation of NRF2 (Ahuja et al., 2021). In MPTP-mice, HPPE treatment significantly blocked accumulation of oxidative stress markers, reduced neuro-inflammation and correspondingly, it increased neuroprotective genes (Ahuja et al., 2021) (Table 3).

**TABLE 3:** BACH1 inhibitors induce the NRF2 pathway in neurodegenerative conditions. This table details how BACH1 inhibiting compounds leads to the activation of NRF2, promoting antioxidant responses and providing neuroprotection. The table includes data from pre-clinical models of AD and PD, showing reductions in oxidative stress, decreased neuronal loss, and improvements in disease-related symptoms following treatment with BACH1 inhibitors.

Disease	Experimental model	Treatment regimen	Major outcomes	References
Alzheimer’s Disease *in vitro*	BV2 microglia activated by APN	Heme (5 μM)	Bach1 inhibition increased the basal expression of Nrf2-antioxidant target genes	Zhang et al. (2021)
	BV2 microglia stimulated by LPS	DMH-CBD (1, 5, and 10 μM)	Upregulated expression of genes related to oxidative stress including HMOX-1 and simultaneous downregulation of expression of pro-inflammatory genes induced by LPS	Juknat et al. (2016)
	BV2 microglia N18TG2 cells stressed by rotenone	CBD (10 μM)	Upregulation of NRF2 gene expression and antioxidant HMOX-1; downregulation of pro-inflammatory IL-6	Duvigneau et al. (2020)
Alzheimer’s Disease *in vivo*	Adult male Sprague-Dawley rats inoculated with human Aβ_1-42_	CBD (10 mg/kg)	CBD treatment decreased Aβ-induced neuroinflammation and facilitates hippocampal neurogenesis	Esposito et al. (2011)
Parkinson’s Disease *In vivo*	C57BL/6J mice in the MPTP-PD model	HPPE (5, 10 and 50 mg/kg) oral gavage	Bach1 nuclear export significantly blocked accumulation of oxidative stress marker 3-nitrotyrosine, numbers of reactive microglia and astrocytes in the SNpc were reduced accompanied by a corresponding increase in neuroprotective genes	Ahuja et al. (2021)

In conclusion, despite a limited number of studies, the targeting BACH1 has shown promise in achieving disease modification for NDD.

## Molecular challenges in activating the NRF2 antioxidant response

Even though KEAP1 shows potential as a molecular target for the rescue of NRF2 defects, targeting KEAP1 for therapeutic purposes remains a challenge. First, KEAP1 has been shown to have complex interactions with various other proteins besides NRF2 (Hushpulian et al., 2021) and concerns about potential side effects arise from the diverse array of cellular processes covered by KEAP1 interactor proteins, involving DNA replication, cytoskeletal dynamics, transcription, and apoptosis. Secondly, small molecules designed to displace KEAP1 from NRF2 primarily target the Kelch domain in KEAP1. However, it is crucial to note that KEAP1 is only one member of a larger group of 42 Kelch-BTB proteins, each exerting essential roles in biological processes, primarily through substrate ubiquitination (Shi et al., 2019). Consequently, a small molecule targeting the Kelch domain of KEAP1 is highly likely to cross-react with the other proteins containing Kelch domains.

**FIGURE 4:**
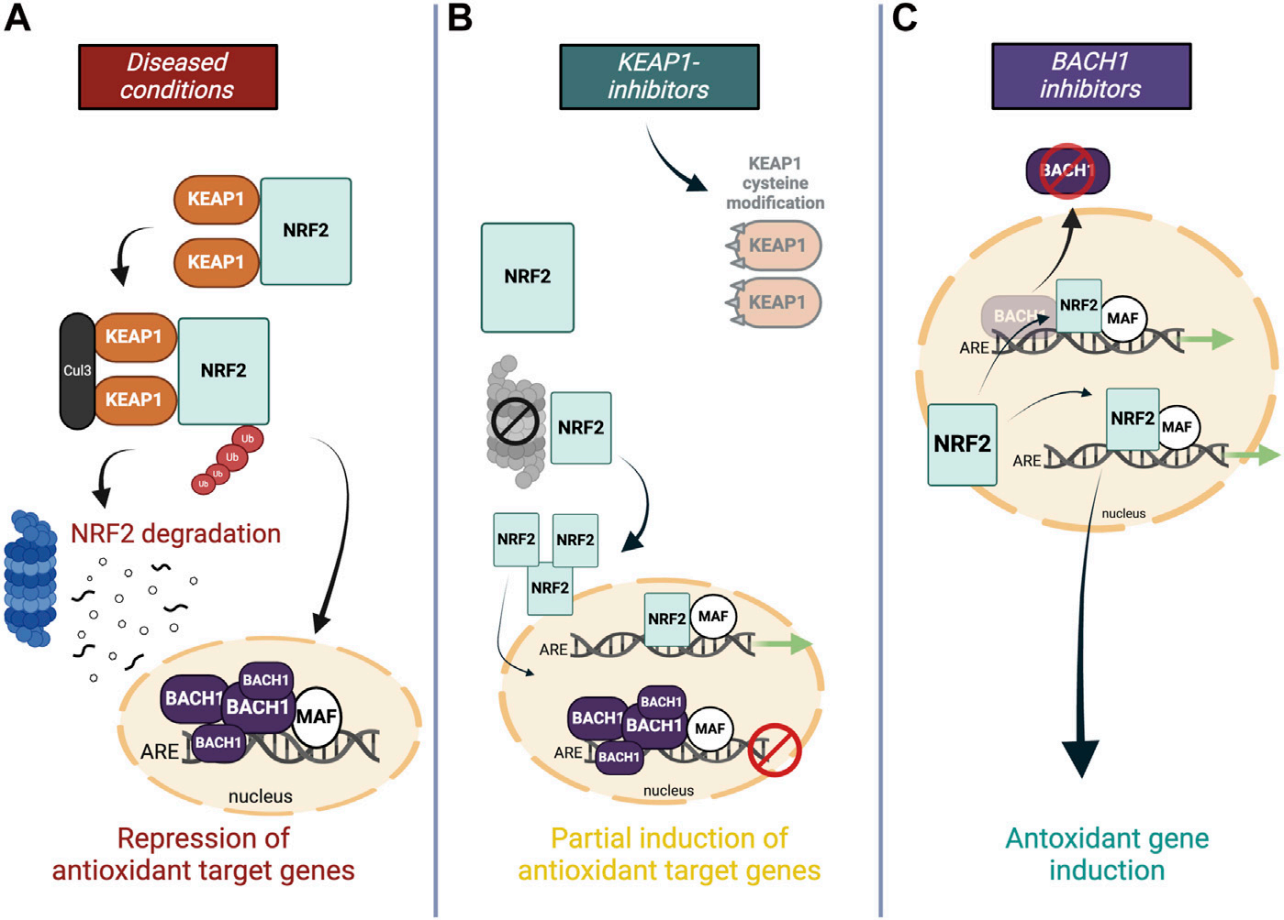
Molecular targets within the NRF2 pathway **(A)** In oxidative stress conditions, NRF2 signaling is hampered by ongoing proteasome-dependent degradation in the cytoplasm through KEAP1, and antagonistic repression of NRF2’s DNA-binding site in the nucleus by BACH1. **(B)** Most NRF2 activators modify or replace KEAP1 to facilitate NRF2’s translocation into the nucleus, however, higher levels of NRF2 are accompanied by an increased negative feedback response, and heightened BACH1 levels in the nucleus impede the full antioxidant gene expression by occupying the ARE binding site. **(C)** Downregulating transcriptional inhibitor BACH1 allows the induction of NRF2-induced antioxidant genes without activating negative feedback regulators.

Numerous studies on neurodegenerative diseases have shown that NRF2 stabilizes under chronic oxidative stress and inflammation but it still fails to increase the antioxidant genetic program to the level needed to fight against the ongoing oxidative stress. One explanation for this is attributed to the feedback regulation within the pathway–continuous NRF2 activation triggers the cellular program to express NRF2 transcriptional repressors (Figures 4A,B). A study demonstrated that the induction of NRF2-mediated cytoprotective responses is diminished in cells from older adults, which is accompanied by an increase of its transcriptional repressor BACH1 (Zhou et al., 2018). Conversely, the expression of NRF2-target genes was shown to be enhanced in silenced BACH1 cells from older subjects (Zhang et al., 2019). Hence, BACH1 provides feedback mechanisms to counteract activation of NRF2 meaning that NRF2 protein stabilization is accompanied by an increase in expression levels of its own epigenetic repressor BACH1 (Zhou et al., 2018; Zhang et al., 2019). Indeed, upregulated BACH1 levels have been reported in post-mortem PD brains as well as in preclinical disease models and further associated with repression of the expression of NRF2 protective genes (Zhou et al., 2018; Ahuja et al., 2021). BACH1 knockout mice have been reported to be protected against MPTP-induced DA neurotoxicity and associated oxidative damage and neuroinflammation, which was substantiated by functional genomics data that demonstrated increased BACH1-targeted pathways (Ahuja et al., 2021). Considering the above and BACH1s vital role in maintaining cellular redox homeostasis, the transcription factor presents a novel molecular target which intrinsically modulates the NRF2 pathway without breaking its negative-feedback loop (Figure 4C).

## Novel drug modalities for NRF2 activation

Up to date, the only NRF2 activators in clinical use are DMF for MS (Rosito et al., 2020), and more recently Omaveloxolone, a semisynthetic terpenoid with potent NRF2-activating effects gained FDA approval in Februrary 2023 for the treatment of Friederich’s ataxia (Boesch and Indelicato, 2024), a rare inherited neurodegenerative disease. Despite its recent approval, the mechanism of action of Omevaloxolone is still unknown, although it is thought to act through KEAP1 inhibition.

However, most NRF2 activators are of electrophilic nature and exhibit unpredictable poly-pharmacology. This was confirmed in one MS mouse model, which showed that DMF-mediated protection against acute inflammatory autoimmune encephalomyelitis was observed in both NRF2-proficient and NRF2-deficient animals (Schulze-Topphoff et al., 2016) and likewise, SFN has been demonstrated to have hundreds of molecular targets next to KEAP1 (Clulow et al., 2017). Due to that, many have failed to receive FDA approval. To overcome the problem of unknown and unpredictable off-target effects of electrophiles, small molecule protein-protein inhibitors (PPI) have been developed (Li et al., 2020). Those non-covalent compounds are highly specific and since they bind reversibly, they may exhibit a better safety profile (Abed et al., 2015). However, existing small molecules lack *in vivo* activity (Zhuang et al., 2017), potentially because they are highly polar and hardly able to penetrate the BBB. Due to their poor stability and low bioavailability, it is difficult to develop KEAP1-NRF2 PPIs as neurodegenerative drugs.

In contrast, RNA-based therapies have gained interest as a promising modality in treating neurological diseases (Holm et al., 2022) and they demonstrate advantages over conventional small molecule pharmaceuticals. Due to their mode of action being the direct modification, upregulation, or inhibition of transcripts (Juliano and Carver, 2015; Crooke, 2017), RNA-based drugs can be designed to modulate their therapeutic target specifically, rendering them highly valuable for pharmacologic interaction with biomolecules, or entire disease pathways, which are currently considered undruggable with small molecules or monoclonal antibodies. Hence, RNA therapeutics have great potential to be developed as personalized medicine, targeting heterogeneous characteristics of neurodegenerative diseases in patients individually. Since the only information required for their synthesis is the sequence of the target RNA (Kim et al., 2019), screening of drug lead candidates can be performed rapidly, and the availability of commercial vendors allows for a fast and cheap turnaround time. When RNA therapeutics are applied in the brain, they are usually delivered to the CNS through intrathecal injections, ensuring adequate drug levels in disease-relevant areas by circumventing the BBB (Holm et al., 2022). Although this has several advantages, it requires administration in primary care centers and long dosing intervals. However, chemical modifications have improved the drug-like characteristics of RNA modalities (Khvorova and Watts, 2017), rendering them safer and more stable *in vivo*, with half-lives extending over 6 months for Antisense Oligonucleotides (Scoles et al., 2019). Accordingly, RNA drug platforms represent a promising novel modality to make disease-modifying therapies for neurodegenerative diseases a reality.

## Concluding remarks

The transcription factor NRF2 is central to the regulatory network governing cellular defenses against toxic and oxidative insults. When activated, this master regulator and its target genes attenuate numerous processes involved in neurodegeneration, including oxidative stress, neuroinflammation and mitochondrial dysfunction. In contrast to therapeutic interventions based on solely rectifying the pathology of proteinopathies, the stimulation of a pathway governing multiple disease processes holds the potential to modify disease dynamics and progression. Thus, targeting the NRF2-ARE axis can provide long-lasting and comprehensive neuronal protection, rendering it highly attractive for the treatment of neurodegenerative diseases. Presently, the plethora of identified NRF2 activators is of electrophilic nature and targets the reactive cysteine residues of KEAP1. Despite their potential to ameliorate neurodegeneration through upregulation of NRF2 and its target genes, as shown by numerous pre-clinical studies, their molecular off-target effects and concomitant unpredictable side-effects pose an obstacle for clinical trials and approval. On the other hand, PPIs that have evolved to overcome the drawbacks of covalent modifiers lack bioavailability and are currently not applicable for the treatment of neurological disorders. A more recently established target in the antioxidant pathway is BACH1, which serves as a negative regulator of NRF2. By acting as a physiological repressor of NRF2 target genes at the ARE-binding site, knockdown or nuclear exclusion of BACH1 holds promising potential for reactivating the downregulated activity of NRF2 associated with disease. Future challenges to overcome in the development of NRF2 activators as disease modifying treatments include the establishment of compounds with good pharmacokinetic and pharmacodynamic profiles that ensure sustained, brain wide NRF2 activation. In light of this, RNA-based therapies hold greater therapeutic potential than current pharmaceutical designs, and offer the possibility to revolutionize the therapeutic landscape of neurodegenerative diseases.

## Figures and Tables

**Table:** 

**6-OHDA**	6-hydroxydopamine
**AD**	Alzheimer’s Disease
**ALS**	Amyotrophic lateral sclerosis
**ARE**	Antioxidant response element
**ASO**	Antisense oligonucleotide
**ATP**	Adenosine triphosphate
**Aβ**	β-amyloid
**BACH1**	BTB and CNC homology 1
**BBB**	Blood brain barrier
**CNC**	Cap ‘n’ Collar
**CNS**	Central nervous system
**DA**	Dopaminergic
**DAMP**	Danger associated molecular patterns
**DMF**	Dimethyl fumarate
**DNA**	Deoxyribonuclease
**EAE**	Experimental autoimmune encephalomyelitis
**ETC**	Electron transport chain
**FDA**	U.S. Food and Drug Administration
**GSK-3β**	Glycogen synthase kinase-3 beta
**HD**	Huntington’s Disease
**HMOX-1**	Heme oxygenase 1
**HPPE**	N-(2-(2-hydroxyethoxy)ethyl)-1-methyl-2-((6-(trifluoromethyl)benzo[d]thiazol-2-yl)amino)-1H-benzo[d]imidazole-5-carboxamide
**IL**	Interleukin
**KEAP1**	Kelch like ECH associated protein 1
**LPS**	Lipopolysaccharide
**LRRK2**	Leucine-rich repeat kinase 2
**MARE**	Maf recognition element
**MPTP**	1-methyl-4-phenyl-l,2,3,6-tetrahydropyridine
**mRNA**	Messenger RNA
**MS**	Multiple Sclerosis
**mtDNA**	Mitochondrial DNA
**NDD**	Neurodegenerative disease
**NF-κB**	Nuclear factor kappa-light-chain-enhancer of activated B cells
**NOS**	Nitric oxide synthase
**NQO-1**	NAD(P)H quinone dehydrogenase 1
**NRF2**	Nuclear factor erythroid 2–related factor 2
**PD**	Parkinson’s Disease
**PINK1**	PTEN-induced putative kinase 1
**PRX**	Peroxiredoxin
**RNA**	Ribonucleic acid
**RNS**	Reactive nitrogen species
**ROS**	Reactive oxygen species
**SFN**	Sulforaphane
**sMAF**	Small musculoaponeurotic fibrosarcoma proteins
**SNpc**	Substantia nigra pars compacta
**SOD1**	Superoxide dismutase 1

## References

[B1] Abdalkader M. Lampinen R. Kanninen K. M. Malm T. M. Liddell J. R. (2018). Targeting Nrf2 to suppress ferroptosis and mitochondrial dysfunction in neurodegeneration. Front. Neurosci. 12, 466. 10.3389/fnins.2018.00466 30042655 PMC6048292

[B2] Abe K. Pan L. H. Watanabe M. Kato T. Itoyama Y. (1995). Induction of nitrotyrosine-like immunoreactivity in the lower motor neuron of amyotrophic lateral sclerosis. Neurosci. Lett. 199, 152–154. 10.1016/0304-3940(95)12039-7 8584246

[B3] Abed D. A. Goldstein M. Albanyan H. Jin H. Hu L. (2015). Discovery of direct inhibitors of Keap1–Nrf2 protein–protein interaction as potential therapeutic and preventive agents. Acta Pharm. Sin. B 5, 285–299. 10.1016/J.APSB.2015.05.008 26579458 PMC4629420

[B4] Acar G. Idiman F. Idiman E. Kirkali G. Çakmakçi H. Özakbaş S. (2003). Nitric oxide as an activity marker in multiple sclerosis. J. Neurol. 250, 588–592. 10.1007/s00415-003-1041-0 12736739

[B5] Agrawal M. (2020). “Molecular basis of chronic neurodegeneration,” in Clinical molecular medicine: principles and practice. Editor Kumar D. (Elsevier), 447–460. 10.1016/B978-0-12-809356-6.00026-5

[B6] Ahmadi M. Agah E. Nafissi S. Jaafari M. R. Harirchian M. H. Sarraf P. (2018). Safety and efficacy of nanocurcumin as add-on therapy to riluzole in patients with amyotrophic lateral sclerosis: a pilot randomized clinical trial. Neurotherapeutics 15, 430–438. 10.1007/S13311-018-0606-7 29352425 PMC5935637

[B7] Ahuja M. Kaidery N. A. Attucks O. C. McDade E. Hushpulian D. M. Gaisin A. (2021). Bach1 derepression is neuroprotective in a mouse model of Parkinson’s disease. Proc. Natl. Acad. Sci. U. S. A. 118, e2111643118. 10.1073/pnas.2111643118 34737234 PMC8694049

[B8] Ahuja M. Kaidery N. A. Dutta D. Attucks O. C. Kazakov E. H. Gazaryan I. (2022). Harnessing the therapeutic potential of the nrf2/bach1 signaling pathway in Parkinson’s disease. Antioxidants 11, 1780. 10.3390/antiox11091780 36139853 PMC9495572

[B9] Ahuja M. Kaidery N. A. Yang L. Calingasan N. Smirnova N. Gaisin A. (2016). Distinct Nrf2 signaling mechanisms of fumaric acid esters and their role in neuroprotection against 1-methyl-4-phenyl-1,2,3,6-tetrahydropyridine-induced experimental Parkinson’s-like disease. J. Neurosci. 36, 6332–6351. 10.1523/JNEUROSCI.0426-16.2016 27277809 PMC4899530

[B10] Alam Z. I. Jenner A. Daniel S. E. Lees A. J. Cairns N. Marsden C. D. (1997). Oxidative DNA damage in the parkinsonian brain: an apparent selective increase in 8-hydroxyguanine levels in substantia nigra. J. Neurochem. 69, 1196–1203. 10.1046/J.1471-4159.1997.69031196.X 9282943

[B11] Allan Butterfield D. Boyd-Kimball D. (2018). Oxidative stress, amyloid-β peptide, and altered key molecular pathways in the pathogenesis and progression of Alzheimer’s disease. J. Alzheimer’s Dis. 62, 1345–1367. 10.3233/JAD-170543 29562527 PMC5870019

[B12] Amoroso R. Maccallini C. Bellezza I. (2023). Activators of Nrf2 to counteract neurodegenerative diseases. Antioxidants (Basel) 12, 778. 10.3390/antiox12030778 36979026 PMC10045503

[B13] An Y. W. Jhang K. A. Woo S. Y. Kang J. L. Chong Y. H. (2016). Sulforaphane exerts its anti-inflammatory effect against amyloid-β peptide via STAT-1 dephosphorylation and activation of Nrf2/HO-1 cascade in human THP-1 macrophages. Neurobiol. Aging 38, 1–10. 10.1016/J.NEUROBIOLAGING.2015.10.016 26827637

[B14] Andersen P. M. Al-Chalabi A. (2011). Clinical genetics of amyotrophic lateral sclerosis: what do we really know? Nat. Rev. Neurol. 7, 603–615. 10.1038/NRNEUROL.2011.150 21989245

[B15] Bahn G. Park J. S. Yun U. J. Lee Y. J. Choi Y. Park J. S. (2019). NRF2/ARE pathway negatively regulates BACE1 expression and ameliorates cognitive deficits in mouse Alzheimer’s models. Proc. Natl. Acad. Sci. U. S. A. 116, 12516–12523. 10.1073/pnas.1819541116 31164420 PMC6589670

[B16] Ban M. Elson J. Walton A. Turnbull D. Compston A. Chinnery P. (2008). Investigation of the role of mitochondrial DNA in multiple sclerosis susceptibility. PLoS One 3, e2891. 10.1371/JOURNAL.PONE.0002891 18682780 PMC2494944

[B17] Bao B. Zhang M. Q. Chen Z. Y. Wu X. B. Xia Z. B. Chai J. Y. (2019). Sulforaphane prevents PC12 cells from oxidative damage via the Nrf2 pathway. Mol. Med. Rep. 19, 4890–4896. 10.3892/MMR.2019.10148 31059012 PMC6522909

[B18] Beal M. F. Ferrante R. J. Browne S. E. Matthews R. T. Kowall N. W. Brown R. H. (1997). Increased 3-nitrotyrosine in both sporadic and familial amyotrophic lateral sclerosis. Ann. Neurol. 42, 644–654. 10.1002/ANA.410420416 9382477

[B19] Béraud D. Maguire-Zeiss K. A. (2012). Misfolded α-synuclein and toll-like receptors: therapeutic targets for Parkinson’s disease. Park. Relat. Disord. 18, S17–S20. 10.1016/S1353-8020(11)70008-6 PMC3500631 22166424

[B20] Blagov A. V. Sukhorukov V. N. Orekhov A. N. Sazonova M. A. Melnichenko A. A. (2022). Significance of mitochondrial dysfunction in the progression of multiple sclerosis. Int. J. Mol. Sci. 23, 12725. 10.3390/IJMS232112725 36361513 PMC9653869

[B21] Blasco H. Garcon G. Patin F. Veyrat-Durebex C. Boyer J. Devos D. (2017). Panel of oxidative stress and inflammatory biomarkers in ALS: a pilot study. Can. J. Neurological Sci. 44, 90–95. 10.1017/CJN.2016.284 27774920

[B22] Blokhin A. Vyshkina T. Komoly S. Kalman B. (2008). Variations in mitochondrial DNA copy numbers in MS brains. J. Mol. Neurosci. 35, 283–287. 10.1007/s12031-008-9115-1 18566918

[B23] Boesch S. Indelicato E. (2024). Approval of omaveloxolone for Friedreich ataxia. Nat. Rev. Neurol. 20, 313–314. 10.1038/s41582-024-00957-9 38570703

[B24] Bomprezzi R. (2015). Dimethyl fumarate in the treatment of relapsing–remitting multiple sclerosis: an overview. Ther. Adv. Neurol. Disord. 8, 20–30. 10.1177/1756285614564152 25584071 PMC4286944

[B25] Brackhan M. Arribas-Blazquez M. Lastres-Becker I. (2023). Aging, NRF2, and tau: a perfect match for neurodegeneration? Antioxidants 12, 1564. 10.3390/ANTIOX12081564 37627559 PMC10451380

[B26] Bresciani G. Manai F. Davinelli S. Tucci P. Saso L. Amadio M. (2023). Novel potential pharmacological applications of dimethyl fumarate—an overview and update. Front. Pharmacol. 14, 1264842. 10.3389/fphar.2023.1264842 37745068 PMC10512734

[B27] Brockmann K. Apel A. Schulte C. Schneiderhan-Marra N. Pont-Sunyer C. Vilas D. (2016). Inflammatory profile in LRRK2-associated prodromal and clinical PD. J. Neuroinflammation 13, 122. 10.1186/s12974-016-0588-5 27220776 PMC4879729

[B28] Brodacki B. Staszewski J. Toczyłowska B. Kozłowska E. Drela N. Chalimoniuk M. (2008). Serum interleukin (IL-2, IL-10, IL-6, IL-4), TNFalpha, and INFgamma concentrations are elevated in patients with atypical and idiopathic parkinsonism. Neurosci. Lett. 441, 158–162. 10.1016/J.NEULET.2008.06.040 18582534

[B29] Campolo M. Casili G. Biundo F. Crupi R. Cordaro M. Cuzzocrea S. (2017). The neuroprotective effect of dimethyl fumarate in an MPTP-mouse model of Parkinson’s disease: involvement of reactive oxygen species/nuclear factor-κb/nuclear transcription factor related to NF-E2. Antioxid. Redox Signal 27, 453–471. 10.1089/ARS.2016.6800 28006954 PMC5564046

[B30] Campolo M. Casili G. Lanza M. Filippone A. Paterniti I. Cuzzocrea S. (2018). Multiple mechanisms of dimethyl fumarate in amyloid β-induced neurotoxicity in human neuronal cells. J. Cell Mol. Med. 22, 1081–1094. 10.1111/JCMM.13358 28990726 PMC5783882

[B31] Casares L. García V. Garrido-Rodríguez M. Millán E. Collado J. A. García-Martín A. (2020). Cannabidiol induces antioxidant pathways in keratinocytes by targeting BACH1. Redox Biol. 28, 101321. 10.1016/J.REDOX.2019.101321 31518892 PMC6742916

[B32] Cassina P. Cassina A. Pehar M. Castellanos R. Gandelman M. De León A. (2008). Mitochondrial dysfunction in sod1g93a-bearing astrocytes promotes motor neuron degeneration: prevention by mitochondrial-targeted antioxidants. J. Neurosci. 28, 4115–4122. 10.1523/JNEUROSCI.5308-07.2008 18417691 PMC3844766

[B33] Chen P. C. Vargas M. R. Pani A. K. Smeyne R. J. Johnson D. A. Kan Y. W. (2009). Nrf2-mediated neuroprotection in the MPTP mouse model of Parkinson’s disease: critical role for the astrocyte. Proc. Natl. Acad. Sci. U. S. A. 106, 2933–2938. 10.1073/pnas.0813361106 19196989 PMC2650368

[B34] Chen W. J. Du J. K. Hu X. Yu Q. Li D. X. Wang C. N. (2017). Protective effects of resveratrol on mitochondrial function in the hippocampus improves inflammation-induced depressive-like behavior. Physiol. Behav. 182, 54–61. 10.1016/J.PHYSBEH.2017.09.024 28964807

[B35] Chico L. Ienco E. C. Bisordi C. Lo Gerfo A. Petrozzi L. Petrucci A. (2018). Amyotrophic lateral sclerosis and oxidative stress: a double-blind therapeutic trial after curcumin supplementation. CNS Neurol. Disord. Drug Targets 17, 767–779. 10.2174/1871527317666180720162029 30033879

[B36] Chiot A. Zaïdi S. Iltis C. Ribon M. Berriat F. Schiaffino L. (2020). Modifying macrophages at the periphery has the capacity to change microglial reactivity and to extend ALS survival. Nat. Neurosci. 23 (11), 1339–1351. 10.1038/s41593-020-00718-z 33077946

[B37] Choi I. Y. Lee P. Adany P. Hughes A. J. Belliston S. Denney D. R. (2018). *In vivo* evidence of oxidative stress in brains of patients with progressive multiple sclerosis. Multiple Scler. J. 24, 1029–1038. 10.1177/1352458517711568 PMC5711636 28569645

[B38] Chowdhury I. Mo Y. Gao L. Kazi A. Fisher A. B. Feinstein S. I. (2009). Oxidant stress stimulates expression of the human peroxiredoxin 6 gene by a transcriptional mechanism involving an antioxidant response element. Free Radic. Biol. Med. 46, 146–153. 10.1016/j.freeradbiomed.2008.09.027 18973804 PMC2646855

[B39] Clulow J. A. Storck E. M. Lanyon-Hogg T. Kalesh K. A. Jones L. H. Tate E. W. (2017). Competition-based, quantitative chemical proteomics in breast cancer cells identifies new target profiles for sulforaphane. Chem. Commun. 53, 5182–5185. 10.1039/C6CC08797C PMC6034444 28439590

[B40] Colamartino M. Duranti G. Ceci R. Sabatini S. Testa A. Cozzi R. (2018). A multi-biomarker analysis of the antioxidant efficacy of Parkinson’s disease therapy. Toxicol. Vitro 47, 1–7. 10.1016/J.TIV.2017.10.020 29080800

[B41] Crooke S. T. (2017). Molecular mechanisms of Antisense oligonucleotides. Nucleic Acid. Ther. 27, 70–77. 10.1089/NAT.2016.0656 28080221 PMC5372764

[B42] Cuadrado A. Rojo A. I. Wells G. Hayes J. D. Cousin S. P. Rumsey W. L. (2019). Therapeutic targeting of the NRF2 and KEAP1 partnership in chronic diseases. Nat. Rev. Drug Discov. 18 (4), 295–317. 10.1038/s41573-018-0008-x 30610225

[B43] Cummings J. (2023). Anti-amyloid monoclonal antibodies are transformative treatments that redefine alzheimer’s disease therapeutics. Drugs 83, 569–576. 10.1007/S40265-023-01858-9 37060386 PMC10195708

[B44] Deng C. Tao R. Yu S. Z. Jin H. (2012a). Inhibition of 6-hydroxydopamine-induced endoplasmic reticulum stress by sulforaphane through the activation of Nrf2 nuclear translocation. Mol. Med. Rep. 6, 215–219. 10.3892/mmr.2012.894 22552270

[B45] Deng C. Tao R. Yu S. Z. Jin H. (2012b). Sulforaphane protects against 6-hydroxydopamine-induced cytotoxicity by increasing expression of heme oxygenase-1 in a PI3K/Akt-dependent manner. Mol. Med. Rep. 5, 847–851. 10.3892/MMR.2011.731 22200816

[B46] De Riccardis L. Buccolieri A. Muci M. Pitotti E. De Robertis F. Trianni G. (2018). Copper and ceruloplasmin dyshomeostasis in serum and cerebrospinal fluid of multiple sclerosis subjects. Biochimica Biophysica Acta (BBA) - Mol. Basis Dis. 1864, 1828–1838. 10.1016/J.BBADIS.2018.03.007 29524632

[B47] d’Errico P. Ziegler-Waldkirch S. Aires V. Hoffmann P. Mezö C. Erny D. (2021). Microglia contribute to the propagation of Aβ into unaffected brain tissue. Nat. Neurosci. 25 (1), 20–25. 10.1038/s41593-021-00951-0 34811521 PMC8737330

[B48] Deture M. A. Dickson D. W. (2019). The neuropathological diagnosis of Alzheimer’s disease. Mol. Neurodegener. 14 (1), 32–18. 10.1186/S13024-019-0333-5 31375134 PMC6679484

[B49] Devi L. Raghavendran V. Prabhu B. M. Avadhani N. G. Anandatheerthavarada H. K. (2008). Mitochondrial import and accumulation of alpha-synuclein impair complex I in human dopaminergic neuronal cultures and Parkinson disease brain. J. Biol. Chem. 283, 9089–9100. 10.1074/JBC.M710012200 18245082 PMC2431021

[B50] Dhakshinamoorthy S. Jain A. K. Bloom D. A. Jaiswal A. K. (2005). Bach1 competes with Nrf2 leading to negative regulation of the antioxidant response element (ARE)-mediated NAD(P)H:quinone oxidoreductase 1 gene expression and induction in response to antioxidants. J. Biol. Chem. 280, 16891–16900. 10.1074/JBC.M500166200 15734732

[B51] Di Filippo M. Chiasserini D. Tozzi A. Picconi B. Calabresi P. (2010). Mitochondria and the link between neuroinflammation and neurodegeneration. J. Alzheimers Dis. 20 (Suppl. 2), S369–S379. 10.3233/JAD-2010-100543 20463396

[B52] Ding C. Wu Y. Chen X. Chen Y. Wu Z. Lin Z. (2022). Global, regional, and national burden and attributable risk factors of neurological disorders: the Global Burden of Disease study 1990–2019. Front. Public Health 10, 952161. 10.3389/fpubh.2022.952161 36523572 PMC9745318

[B53] Duvigneau J. C. Trovato A. Müllebner A. Miller I. Krewenka C. Krenn K. (2020). Cannabidiol protects dopaminergic neurons in mesencephalic cultures against the complex I inhibitor rotenone via modulation of heme oxygenase activity and bilirubin. Antioxidants 9, 135. 10.3390/ANTIOX9020135 32033040 PMC7070382

[B54] Dysken M. W. Sano M. Asthana S. Vertrees J. E. Pallaki M. Llorente M. (2014). Effect of Vitamin E and memantine on functional decline in alzheimer disease: the TEAM-AD VA cooperative randomized trial. JAMA J. Am. Med. Assoc. 311, 33–44. 10.1001/JAMA.2013.282834 PMC4109898 24381967

[B55] Erkkinen M. G. Kim M. O. Geschwind M. D. (2018). Clinical neurology and epidemiology of the major neurodegenerative diseases. Cold Spring Harb. Perspect. Biol. 10, a033118. 10.1101/cshperspect.a033118 28716886 PMC5880171

[B56] Esposito G. Scuderi C. Valenza M. Togna G. I. Latina V. de Filippis D. (2011). Cannabidiol reduces Aβ-induced neuroinflammation and promotes hippocampal neurogenesis through PPARγ involvement. PLoS One 6, e28668. 10.1371/JOURNAL.PONE.0028668 22163051 PMC3230631

[B57] Fakhri S. Pesce M. Patruno A. Moradi S. Z. Iranpanah A. Farzaei M. H. (2020). Attenuation of nrf2/keap1/ARE in alzheimer’s disease by plant secondary metabolites: a mechanistic review. Molecules 25, 4926. 10.3390/MOLECULES25214926 33114450 PMC7663041

[B58] Filippi M. Bar-Or A. Piehl F. Preziosa P. Solari A. Vukusic S. (2018). Multiple sclerosis. Nat. Rev. Dis. Prim. 4 (1), 43–27. 10.1038/s41572-018-0041-4 30410033

[B59] Forte M. Gold B. G. Marracci G. Chaudhary P. Basso E. Johnsen D. (2007). Cyclophilin D inactivation protects axons in experimental autoimmune encephalomyelitis, an animal model of multiple sclerosis. Proc. Natl. Acad. Sci. U. S. A. 104, 7558–7563. 10.1073/pnas.0702228104 17463082 PMC1857227

[B60] Fox R. J. Miller D. H. Phillips J. T. Hutchinson M. Havrdova E. Kita M. (2012). Placebo-controlled phase 3 study of oral BG-12 or glatiramer in multiple sclerosis. N. Engl. J. Med. 367, 1087–1097. 10.1056/NEJMoa1206328 22992072

[B61] Fu M. H. Wu C. W. Lee Y. C. Hung C. Y. Chen I. C. Wu K. L. H. (2018). Nrf2 activation attenuates the early suppression of mitochondrial respiration due to the α-synuclein overexpression. Biomed. J. 41, 169–183. 10.1016/J.BJ.2018.02.005 30080657 PMC6138761

[B62] Ghasemi M. Brown R. H. (2018). Genetics of amyotrophic lateral sclerosis. Cold Spring Harb. Perspect. Med. 8, a024125. 10.1101/CSHPERSPECT.A024125 28270533 PMC5932579

[B63] Gilgun-Sherki Y. Melamed E. Offen D. (2004). The role of oxidative stress in the pathogenesis of multiple sclerosis: the need for effective antioxidant therapy. J. Neurol. 251, 261–268. 10.1007/s00415-004-0348-9 15015004

[B64] Gillard G. O. Collette B. Anderson J. Chao J. Scannevin R. H. Huss D. J. (2015). DMF, but not other fumarates, inhibits NF-κB activity *in vitro* in an Nrf2-independent manner. J. Neuroimmunol. 283, 74–85. 10.1016/J.JNEUROIM.2015.04.006 26004161

[B65] Gold R. Kappos L. Arnold D. L. Bar-Or A. Giovannoni G. Selmaj K. (2012). Placebo-controlled phase 3 study of oral BG-12 for relapsing multiple sclerosis. N. Engl. J. Med. 367, 1098–1107. 10.1056/NEJMoa1114287 22992073

[B66] Gold R. Linington C. Lassmann H. (2006). Understanding pathogenesis and therapy of multiple sclerosis via animal models: 70 years of merits and culprits in experimental autoimmune encephalomyelitis research. Brain 129, 1953–1971. 10.1093/BRAIN/AWL075 16632554

[B67] Gumeni S. Papanagnou E. D. Manola M. S. Trougakos I. P. (2021). Nrf2 activation induces mitophagy and reverses Parkin/Pink1 knock down-mediated neuronal and muscle degeneration phenotypes. Cell Death Dis. 12, 671. 10.1038/S41419-021-03952-W 34218254 PMC8254809

[B68] Hammer A. Waschbisch A. Kuhbandner K. Bayas A. Lee D. H. Duscha A. (2018). The NRF2 pathway as potential biomarker for dimethyl fumarate treatment in multiple sclerosis. Ann. Clin. Transl. Neurol. 5, 668–676. 10.1002/ACN3.553 29928650 PMC5989754

[B69] Harding O. Holzer E. Riley J. F. Martens S. Holzbaur E. L. F. (2023). Damaged mitochondria recruit the effector NEMO to activate NF-κB signaling. Mol. Cell 83, 3188–3204.e7. 10.1016/j.molcel.2023.08.005 37683611 PMC10510730

[B70] Hayashi G. Jasoliya M. Sahdeo S. Saccà F. Pane C. Filla A. (2017). Dimethyl fumarate mediates Nrf2-dependent mitochondrial biogenesis in mice and humans. Hum. Mol. Genet. 26, 2864–2873. 10.1093/hmg/ddx167 28460056 PMC6251607

[B71] Hayashi T. Ishimori C. Takahashi-Niki K. Taira T. Kim Y. C. Maita H. (2009). DJ-1 binds to mitochondrial complex I and maintains its activity. Biochem. Biophys. Res. Commun. 390, 667–672. 10.1016/J.BBRC.2009.10.025 19822128

[B72] Hemmati S. Sadeghi M. A. Yousefi-Manesh H. Eslamiyeh M. Vafaei A. Foroutani L. (2020). Protective effects of Leukadherin1 in a rat model of targeted experimental autoimmune encephalomyelitis (EAE): possible role of P47phox and MDA downregulation. J. Inflamm. Res. 13, 411–420. 10.2147/JIR.S258991 32821147 PMC7423460

[B73] Heneka M. T. Kummer M. P. Stutz A. Delekate A. Schwartz S. Vieira-Saecker A. (2013). NLRP3 is activated in Alzheimer’s disease and contributes to pathology in APP/PS1 mice. Nature 493, 674–678. 10.1038/NATURE11729 23254930 PMC3812809

[B74] Higgins C. M. J. Jung C. Xu Z. (2003). ALS-associated mutant SODIG93A causes mitochondrial vacuolation by expansion of the intermembrane space by involvement of SODI aggregation and peroxisomes. BMC Neurosci. 4, 1–14. 10.1186/1471-2202-4-16/FIGURES/10 12864925 PMC169170

[B75] Hindle J. V. (2010). Ageing, neurodegeneration and Parkinson’s disease. Age Ageing 39, 156–161. 10.1093/AGEING/AFP223 20051606

[B76] Hoang T. T. Johnson D. A. Raines R. T. Johnson J. A. (2019). Angiogenin activates the astrocytic Nrf2/antioxidant-response element pathway and thereby protects murine neurons from oxidative stress. J. Biol. Chem. 294, 15095–15103. 10.1074/JBC.RA119.008491 31431502 PMC6791309

[B77] Holm A. Hansen S. N. Klitgaard H. Kauppinen S. (2022). Clinical advances of RNA therapeutics for treatment of neurological and neuromuscular diseases. RNA Biol. 19, 594–608. 10.1080/15476286.2022.2066334 35482908 PMC9067473

[B79] Huang F. (2021). Ursodeoxycholic acid as a potential alternative therapeutic approach for neurodegenerative disorders: effects on cell apoptosis, oxidative stress and inflammation in the brain. Brain Behav. Immun. Health 18, 100348. 10.1016/J.BBIH.2021.100348 34632427 PMC7611783

[B80] Hui C. K. Dedkova E. N. Montgomery C. Cortopassi G. (2021). Dimethyl fumarate dose-dependently increases mitochondrial gene expression and function in muscle and brain of Friedreich’s ataxia model mice. Hum. Mol. Genet. 29, 3954–3965. 10.1093/hmg/ddaa282 33432356 PMC8485216

[B81] Hushpulian D. M. Ammal Kaidery N. Ahuja M. Poloznikov A. A. Sharma S. M. Gazaryan I. G. (2021). Challenges and limitations of targeting the keap1-nrf2 pathway for neurotherapeutics: bach1 de-repression to the rescue. Front. Aging Neurosci. 13, 162. 10.3389/fnagi.2021.673205 PMC8060438 33897412

[B82] Ikram M. Muhammad T. Rehman S. U. Khan A. Jo M. G. Ali T. (2019). Hesperetin confers neuroprotection by regulating nrf2/TLR4/NF-κB signaling in an Aβ mouse model. Mol. Neurobiol. 56, 6293–6309. 10.1007/s12035-019-1512-7 30756299

[B83] Im J. Y. Lee K. W. Woo J. M. Junn E. Mouradian M. M. (2012). DJ-1 induces thioredoxin 1 expression through the Nrf2 pathway. Hum. Mol. Genet. 21, 3013–3024. 10.1093/HMG/DDS131 22492997 PMC3373246

[B84] Isik S. Yeman Kiyak B. Akbayir R. Seyhali R. Arpaci T. (2023). Microglia mediated neuroinflammation in Parkinson’s disease. Cells 12, 1012. 10.3390/CELLS12071012 37048085 PMC10093562

[B85] Jakimovski D. Bittner S. Zivadinov R. Morrow S. A. Benedict R. H. Zipp F. (2024). Multiple sclerosis. Lancet 403, 183–202. 10.1016/S0140-6736(23)01473-3 37949093

[B86] Jazwa A. Rojo A. I. Innamorato N. G. Hesse M. Ferná Ndez-Ruiz J. Cuadrado A. (2011). Pharmacological targeting of the transcription factor Nrf2 at the basal ganglia provides disease modifying therapy for experimental parkinsonism. Antioxid. Redox Signal 14, 2347–2360. 10.1089/ars.2010.3731 21254817

[B88] Ji M. H. Yong J. L. So Y. L. Eun M. K. Moon Y. Ha W. K. (2007). Protective effect of sulforaphane against dopaminergic cell death. J. Pharmacol. Exp. Ther. 321, 249–256. 10.1124/JPET.106.110866 17259450

[B89] Jing X. Shi H. Zhang C. Ren M. Han M. Wei X. (2015). Dimethyl fumarate attenuates 6-OHDA-induced neurotoxicity in SH-SY5Y cells and in animal model of Parkinson’s disease by enhancing Nrf2 activity. Neuroscience 286, 131–140. 10.1016/J.NEUROSCIENCE.2014.11.047 25449120

[B90] Johnson D. A. Amirahmadi S. Ward C. Fabry Z. Johnson J. A. (2010). The absence of the pro-antioxidant transcription factor Nrf2 exacerbates experimental autoimmune encephalomyelitis. Toxicol. Sci. 114, 237–246. 10.1093/TOXSCI/KFP274 19910389 PMC2902921

[B91] Juknat A. Kozela E. Kaushansky N. Mechoulam R. Vogel Z. (2016). Anti-inflammatory effects of the cannabidiol derivative dimethylheptyl-cannabidiol - studies in BV-2 microglia and encephalitogenic T cells. J. Basic Clin. Physiol. Pharmacol. 27, 289–296. 10.1515/jbcpp-2015-0071 26540221

[B92] Juliano R. L. Carver K. (2015). Cellular uptake and intracellular trafficking of oligonucleotides. Adv. Drug Deliv. Rev. 87, 35–45. 10.1016/J.ADDR.2015.04.005 25881722 PMC4504789

[B93] Kalia L. V. Lang A. E. (2015). Parkinson’s disease. Lancet 386, 896–912. 10.1016/S0140-6736(14)61393-3 25904081

[B94] Kalinowska-Łyszczarz A. Szczuciński A. Pawlak M. A. Losy J. (2011). Clinical study on CXCL13, CCL17, CCL20 and IL-17 as immune cell migration navigators in relapsing-remitting multiple sclerosis patients. J. Neurol. Sci. 300, 81–85. 10.1016/J.JNS.2010.09.026 20947098

[B95] Karapetyan G. Fereshetyan K. Harutyunyan H. Yenkoyan K. (2022). The synergy of β amyloid 1-42 and oxidative stress in the development of Alzheimer’s disease-like neurodegeneration of hippocampal cells. Sci. Rep. 12 (1), 17883. 10.1038/s41598-022-22761-5 36284177 PMC9596457

[B96] Katsuoka F. Yamamoto M. (2016). Small maf proteins (MafF, MafG, MafK): history, structure and function. Gene 586, 197–205. 10.1016/J.GENE.2016.03.058 27058431 PMC4911266

[B97] Keeney P. M. Xie J. Capaldi R. A. Bennett J. P. (2006). Parkinson’s disease brain mitochondrial complex I has oxidatively damaged subunits and is functionally impaired and misassembled. J. Neurosci. 26, 5256–5264. 10.1523/JNEUROSCI.0984-06.2006 16687518 PMC6674236

[B98] Khaibullin T. Ivanova V. Martynova E. Cherepnev G. Khabirov F. Granatov E. (2017). Elevated levels of proinflammatory cytokines in cerebrospinal fluid of multiple sclerosis patients. Front. Immunol. 8, 531. 10.3389/FIMMU.2017.00531 28572801 PMC5435759

[B99] Khan A. Ali T. Rehman S. U. Khan M. S. Alam S. I. Ikram M. (2018). Neuroprotective effect of quercetin against the detrimental effects of LPS in the adult mouse brain. Front. Pharmacol. 9, 1383. 10.3389/fphar.2018.01383 30618732 PMC6297180

[B100] Kharel S. Ojha R. (2023). Future of monoclonal antibody therapy in Parkinson’s disease. Ann. Neurosci. 30, 8–10. 10.1177/09727531221136349 37313331 PMC10259150

[B101] Khvorova A. Watts J. K. (2017). The chemical evolution of oligonucleotide therapies of clinical utility. Nat. Biotechnol. 35, 238–248. 10.1038/NBT.3765 28244990 PMC5517098

[B102] Kim H. V. Kim H. Y. Ehrlich H. Y. Choi S. Y. Kim D. J. Kim Y. S. (2013). Amelioration of Alzheimer’s disease by neuroprotective effect of sulforaphane in animal model. Amyloid 20, 7–12. 10.3109/13506129.2012.751367 23253046

[B103] Kim J. Hu C. Moufawad El Achkar C. Black L. E. Douville J. Larson A. (2019). Patient-customized oligonucleotide therapy for a rare genetic disease. N. Engl. J. Med. 381, 1644–1652. 10.1056/NEJMOA1813279 31597037 PMC6961983

[B104] Kirby J. Halligan E. Baptista M. J. Allen S. Heath P. R. Holden H. (2005). Mutant SOD1 alters the motor neuronal transcriptome: implications for familial ALS. Brain 128, 1686–1706. 10.1093/BRAIN/AWH503 15872021

[B105] Kobayashi A. Kang M.-I. Okawa H. Ohtsuji M. Zenke Y. Chiba T. (2004). Oxidative stress sensor Keap1 functions as an adaptor for cul3-based E3 ligase to regulate proteasomal degradation of Nrf2. Mol. Cell Biol. 24, 7130–7139. 10.1128/MCB.24.16.7130-7139.2004 15282312 PMC479737

[B106] Kobayashi E. H. Suzuki T. Funayama R. Nagashima T. Hayashi M. Sekine H. (2016). Nrf2 suppresses macrophage inflammatory response by blocking proinflammatory cytokine transcription. Nat. Commun. 7, 11624. 10.1038/NCOMMS11624 27211851 PMC4879264

[B107] Kovac S. Angelova P. R. Holmström K. M. Zhang Y. Dinkova-Kostova A. T. Abramov A. Y. (2015). Nrf2 regulates ROS production by mitochondria and NADPH oxidase. Biochim. Biophys. Acta 1850, 794–801. 10.1016/J.BBAGEN.2014.11.021 25484314 PMC4471129

[B213] Kovacs G. G. (2019). Molecular pathology of neurodegenerative diseases: principles and practice. J. Clin. Pathol. 72, 725–735. 10.1136/jclinpath-2019-205952 31395625

[B108] Larabee C. M. Desai S. Agasing A. Georgescu C. Wren J. D. Axtell R. C. (2016). Loss of Nrf2 exacerbates the visual deficits and optic neuritis elicited by experimental autoimmune encephalomyelitis. Mol. Vis. 22, 1503–1513. Available at: https://pubmed.ncbi.nlm.nih.gov/28050123/(Accessed September 8, 2023). 28050123 PMC5204460

[B109] Lastres-Becker I. de Lago E. Martínez A. Fernández-Ruiz J. (2022). New statement about NRF2 in amyotrophic lateral sclerosis and frontotemporal dementia. Biomolecules 12, 1200. 10.3390/BIOM12091200 36139039 PMC9496161

[B110] Lastres-Becker I. García-Yagüe A. J. Scannevin R. H. Casarejos M. J. Kügler S. Rábano A. (2016). Repurposing the NRF2 activator dimethyl fumarate as therapy against synucleinopathy in Parkinson’s disease. Antioxid. Redox Signal 25, 61–77. 10.1089/ars.2015.6549 27009601 PMC4943471

[B111] Lastres-Becker I. Ulusoy A. Innamorato N. G. Sahin G. Rábano A. Kirik D. (2012). α-Synuclein expression and Nrf2 deficiency cooperate to aggravate protein aggregation, neuronal death and inflammation in early-stage Parkinson’s disease. Hum. Mol. Genet. 21, 3173–3192. 10.1093/HMG/DDS143 22513881

[B112] Lee C. Park G. H. Lee S. R. Jang J. H. (2013). Attenuation of β-amyloid-induced oxidative cell death by sulforaphane via activation of NF-E2-related factor 2. Oxid. Med. Cell Longev. 2013, 313510. 10.1155/2013/313510 23864927 PMC3705986

[B113] Lee S. Choi B.-R. Kim J. LaFerla F. M. Han Yoon Park J. Han J.-S. (2018). Sulforaphane upregulates the heat shock protein Co-chaperone CHIP and clears amyloid-β and tau in a mouse model of alzheimer’s disease. Mol. Nutr. Food Res. 62, 1800240. 10.1002/MNFR.201800240 29714053

[B214] Leng F. Edison P. (2021). Neuroinflammation and microglial activation in Alzheimer disease: where do we go from here?. Nat. Rev. Neurol. 17, 157–172. 10.1038/s41582-020-00435-y 33318676

[B114] Lev N. Ickowicz D. Melamed E. Offen D. (2008). Oxidative insults induce DJ-1 upregulation and redistribution: implications for neuroprotection. Neurotoxicology 29, 397–405. 10.1016/J.NEURO.2008.01.007 18377993

[B115] Lev N. Roncevich D. Ickowicz D. Melamed E. Offen D. (2006). Role of DJ-1 in Parkinson’s disease. J. Mol. Neurosci. 29, 215–225. 10.1385/jmn:29:3:215 17085780

[B116] Li B. Cui W. Liu J. Li R. Liu Q. Xie X. H. (2013). Sulforaphane ameliorates the development of experimental autoimmune encephalomyelitis by antagonizing oxidative stress and Th17-related inflammation in mice. Exp. Neurol. 250, 239–249. 10.1016/J.EXPNEUROL.2013.10.002 24120440

[B117] Li Q. Xing S. Chen Y. Liao Q. Li Q. Liu Y. (2020). Reasonably activating Nrf2: a long-term, effective and controllable strategy for neurodegenerative diseases. Eur. J. Med. Chem. 185, 111862. 10.1016/J.EJMECH.2019.111862 31735576

[B118] Lim J. H. Kim K. M. Kim S. W. Hwang O. Choi H. J. (2008). Bromocriptine activates NQO1 via Nrf2-PI3K/Akt signaling: novel cytoprotective mechanism against oxidative damage. Pharmacol. Res. 57, 325–331. 10.1016/J.PHRS.2008.03.004 18455424

[B119] Lim J. L. van der Pol S. M. A. Baron W. McCord J. M. de Vries H. E. van Horssen J. (2016). Protandim protects oligodendrocytes against an oxidative insult. Antioxidants 5, 30. 10.3390/ANTIOX5030030 27618111 PMC5039579

[B120] Lu J. Duan W. Guo Y. Jiang H. Li Z. Huang J. (2012). Mitochondrial dysfunction in human TDP-43 transfected NSC34 cell lines and the protective effect of dimethoxy curcumin. Brain Res. Bull. 89, 185–190. 10.1016/J.BRAINRESBULL.2012.09.005 22986236

[B121] Ma Q. (2013). Role of Nrf2 in oxidative stress and toxicity. Annu. Rev. Pharmacol. Toxicol. 53, 401–426. 10.1146/ANNUREV-PHARMTOX-011112-140320 23294312 PMC4680839

[B122] Manczak M. Anekonda T. S. Henson E. Park B. S. Quinn J. Reddy P. H. (2006). Mitochondria are a direct site of A beta accumulation in Alzheimer’s disease neurons: implications for free radical generation and oxidative damage in disease progression. Hum. Mol. Genet. 15, 1437–1449. 10.1093/HMG/DDL066 16551656

[B123] Manczak M. Park B. S. Jung Y. Reddy P. H. (2004). Differential expression of oxidative phosphorylation genes in patients with Alzheimer’s disease: implications for early mitochondrial dysfunction and oxidative damage. Neuromolecular Med. 5, 147–162. 10.1385/NMM:5:2:147 15075441

[B124] Mandal P. K. Roy R. G. Samkaria A. (2022). Oxidative stress: glutathione and its potential to protect methionine-35 of Aβ peptide from oxidation. ACS Omega 7, 27052–27061. 10.1021/acsomega.2c02760 35967059 PMC9366984

[B125] Masci A. Mattioli R. Costantino P. Baima S. Morelli G. Punzi P. (2015). Neuroprotective effect of *Brassica oleracea* sprouts crude juice in a cellular model of alzheimer’s disease. Oxid. Med. Cell Longev. 2015, 781938. 10.1155/2015/781938 26180595 PMC4477226

[B126] Mastroeni D. Khdour O. M. Delvaux E. Nolz J. Olsen G. Berchtold N. (2017). Nuclear but not mitochondrial-encoded oxidative phosphorylation genes are altered in aging, mild cognitive impairment, and Alzheimer’s disease. Alzheimers Dement. 13, 510–519. 10.1016/J.JALZ.2016.09.003 27793643 PMC5967608

[B127] McMahon M. Itoh K. Yamamoto M. Hayes J. D. (2003). Keap1-dependent proteasomal degradation of transcription factor Nrf2 contributes to the negative regulation of antioxidant response element-driven gene expression. J. Biol. Chem. 278, 21592–21600. 10.1074/jbc.M300931200 12682069

[B128] Mehta P. Raymond J. Zhang Y. Punjani R. Han M. Larson T. (2023). Prevalence of amyotrophic lateral sclerosis in the United States, 2018. Amyotroph. Lateral Scler. Front. Degener. 24, 702–708. 10.1080/21678421.2023.2245858 37602649

[B129] Merry T. L. Ristow M. Merry T. L. Ristow M. (2016). Nuclear factor erythroid-derived 2-like 2 (NFE2L2, Nrf2) mediates exercise-induced mitochondrial biogenesis and the anti-oxidant response in mice. J. Physiol. 594, 5195–5207. 10.1113/JP271957 27094017 PMC5023720

[B130] Morgan S. Orrell R. W. (2016). Pathogenesis of amyotrophic lateral sclerosis. Br. Med. Bull. 119, 87–98. 10.1093/BMB/LDW026 27450455

[B131] Morris H. R. Spillantini M. G. Sue C. M. Williams-Gray C. H. (2024). The pathogenesis of Parkinson’s disease. Lancet 403, 293–304. 10.1016/S0140-6736(23)01478-2 38245249

[B132] Morroni F. Sita G. Djemil A. D’Amico M. Pruccoli L. Cantelli-Forti G. (2018). Comparison of adaptive neuroprotective mechanisms of sulforaphane and its interconversion product erucin in *in vitro* and *in vivo* models of Parkinson’s disease. J. Agric. Food Chem. 66, 856–865. 10.1021/acs.jafc.7b04641 29307179

[B133] Morroni F. Tarozzi A. Sita G. Bolondi C. Zolezzi Moraga J. M. Cantelli-Forti G. (2013). Neuroprotective effect of sulforaphane in 6-hydroxydopamine-lesioned mouse model of Parkinson’s disease. Neurotoxicology 36, 63–71. 10.1016/J.NEURO.2013.03.004 23518299

[B134] Mythri R. B. Venkateshappa C. Harish G. Mahadevan A. Muthane U. B. Yasha T. C. (2011). Evaluation of Markers of oxidative stress, antioxidant function and astrocytic proliferation in the striatum and frontal cortex of Parkinson’s disease brains. Neurochem. Res. 36, 1452–1463. 10.1007/s11064-011-0471-9 21484266

[B135] Nagatsu T. Mogi M. Ichinose H. Togari A. (2000). Cytokines in Parkinson’s disease. J. Neural Transm. Suppl., 143–151. 10.1007/978-3-7091-6284-2_12/COVER 11128604

[B136] Navarro A. Boveris A. (2009). Brain mitochondrial dysfunction and oxidative damage in Parkinson’s disease. J. Bioenerg. Biomembr. 41, 517–521. 10.1007/S10863-009-9250-6 19915964

[B137] Niso-Santano M. González-Polo R. A. Bravo-San Pedro J. M. Gómez-Sánchez R. Lastres-Becker I. Ortiz-Ortiz M. A. (2010). Activation of apoptosis signal-regulating kinase 1 is a key factor in paraquat-induced cell death: modulation by the Nrf2/Trx axis. Free Radic. Biol. Med. 48, 1370–1381. 10.1016/J.FREERADBIOMED.2010.02.024 20202476

[B138] Ogawa K. Sun J. Taketani S. Nakajima O. Nishitani C. Sassa S. (2001). Heme mediates derepression of Maf recognition element through direct binding to transcription repressor Bach1. EMBO J. 20, 2835–2843. 10.1093/EMBOJ/20.11.2835 11387216 PMC125477

[B139] Oksanen M. Hyötyläinen I. Trontti K. Rolova T. Wojciechowski S. Koskuvi M. (2020). NF-E2-related factor 2 activation boosts antioxidant defenses and ameliorates inflammatory and amyloid properties in human Presenilin-1 mutated Alzheimer’s disease astrocytes. Glia 68, 589–599. 10.1002/GLIA.23741 31670864 PMC7003860

[B140] Ower A. K. Hadjichrysanthou C. Gras L. Goudsmit J. Anderson R. M. de Wolf F. (2018). Temporal association patterns and dynamics of amyloid-β and tau in Alzheimer’s disease. Eur. J. Epidemiol. 33, 657–666. 10.1007/S10654-017-0326-Z 29071500 PMC6061138

[B141] Paterna J. C. Leng A. Weber E. Feldon J. Büeler H. (2007). DJ-1 and parkin modulate dopamine-dependent behavior and inhibit MPTP-induced nigral dopamine neuron loss in mice. Mol. Ther. 15, 698–704. 10.1038/SJ.MT.6300067 17299411

[B142] Peplow P. V. Martinez B. Gennarelli T. A. (2022). Prevalence, needs, strategies, and risk factors for neurodegenerative diseases. Neuromethods 173, 3–8. 10.1007/978-1-0716-1712-0_1

[B143] Piantadosi C. A. Carraway M. S. Babiker A. Suliman H. B. (2008). Heme oxygenase-1 regulates cardiac mitochondrial biogenesis via nrf2-mediated transcriptional control of nuclear respiratory factor-1. Circ. Res. 103, 1232–1240. AD. 10.1161/01.RES.0000338597.71702.ad 18845810 PMC2694963

[B144] Picca A. Calvani R. Coelho-Júnior H. J. Landi F. Bernabei R. Marzetti E. (2020). Mitochondrial dysfunction, oxidative stress, and neuroinflammation: intertwined roads to neurodegeneration. Antioxidants 9, 647. 10.3390/ANTIOX9080647 32707949 PMC7466131

[B145] Piroli G. G. Manuel A. M. Patel T. Walla M. D. Shi L. Lanci S. A. (2019). Identification of novel protein targets of dimethyl fumarate modification in neurons and astrocytes reveals actions independent of Nrf2 stabilization. Mol. Cell Proteomics 18, 504–519. 10.1074/MCP.RA118.000922 30587509 PMC6398201

[B146] Popescu B. F. G. Pirko I. Lucchinetti C. F. (2013). Pathology of multiple sclerosis: where do we stand? Contin. (Minneap Minn) 19, 901–921. 10.1212/01.CON.0000433291.23091.65 PMC3915566 23917093

[B147] Quinti L. Naidu S. D. Träger U. Chen X. Kegel-Gleason K. Llères D. (2017). KEAP1-modifying small molecule reveals muted NRF2 signaling responses in neural stem cells from Huntington’s disease patients. Proc. Natl. Acad. Sci. U. S. A. 114, E4676–E4685. 10.1073/pnas.1614943114 28533375 PMC5468652

[B148] Ramanathan M. Weinstock-Guttman B. Nguyen L. T. Badgett D. Miller C. Patrick K. (2001). *In vivo* gene expression revealed by cDNA arrays: the pattern in relapsing-remitting multiple sclerosis patients compared with normal subjects. J. Neuroimmunol. 116, 213–219. 10.1016/S0165-5728(01)00308-3 11438176

[B149] Ramsey C. P. Glass C. A. Montgomery M. B. Lindl K. A. Ritson G. P. Chia L. A. (2007). Expression of Nrf2 in neurodegenerative diseases. J. Neuropathol. Exp. Neurol. 66, 75–85. 10.1097/NEN.0B013E31802D6DA9 17204939 PMC2253896

[B150] Rani V. Verma R. Kumar K. Chawla R. (2023). Role of pro-inflammatory cytokines in Alzheimer’s disease and neuroprotective effects of pegylated self-assembled nanoscaffolds. Curr. Res. Pharmacol. Drug Discov. 4, 100149. 10.1016/J.CRPHAR.2022.100149 36593925 PMC9804106

[B151] Ray P. D. Huang B. W. Tsuji Y. (2012). Reactive oxygen species (ROS) homeostasis and redox regulation in cellular signaling. Cell Signal 24, 981–990. 10.1016/J.CELLSIG.2012.01.008 22286106 PMC3454471

[B152] Reale M. Iarlori C. Thomas A. Gambi D. Perfetti B. Di Nicola M. (2009). Peripheral cytokines profile in Parkinson’s disease. Brain Behav. Immun. 23, 55–63. 10.1016/J.BBI.2008.07.003 18678243

[B153] Rehman S. U. Ali T. Alam S. I. Ullah R. Zeb A. Lee K. W. (2019). Ferulic acid rescues LPS-induced neurotoxicity via modulation of the TLR4 receptor in the mouse Hippocampus. Mol. Neurobiol. 56, 2774–2790. 10.1007/s12035-018-1280-9 30058023

[B154] Reichard J. F. Motz G. T. Puga A. (2007). Heme oxygenase-1 induction by NRF2 requires inactivation of the transcriptional repressor BACH1. Nucleic Acids Res. 35, 7074–7086. 10.1093/NAR/GKM638 17942419 PMC2175339

[B155] Rojo A. I. Innamorato N. G. Martín-Moreno A. M. De Ceballos M. L. Yamamoto M. Cuadrado A. (2010). Nrf2 regulates microglial dynamics and neuroinflammation in experimental Parkinson’s disease. Glia 58, 588–598. 10.1002/GLIA.20947 19908287

[B156] Rojo A. I. Pajares M. García-Yagüe A. J. Buendia I. Leuven F. V. Yamamoto M. (2018). Deficiency in the transcription factor NRF2 worsens inflammatory parameters in a mouse model with combined tauopathy and amyloidopathy. Redox Biol. 18, 173–180. 10.1016/j.redox.2018.07.006 30029164 PMC6052199

[B157] Rojo A. I. Pajares M. Rada P. Nuñez A. Nevado-Holgado A. J. Killik R. (2017). NRF2 deficiency replicates transcriptomic changes in Alzheimer’s patients and worsens APP and TAU pathology. Redox Biol. 13, 444–451. 10.1016/J.REDOX.2017.07.006 28704727 PMC5508523

[B158] Rosito M. Testi C. Parisi G. Cortese B. Baiocco P. Di Angelantonio S. (2020). Exploring the use of dimethyl fumarate as microglia modulator for neurodegenerative diseases treatment. Antioxidants 9, 700–722. 10.3390/ANTIOX9080700 32756501 PMC7465338

[B159] Ross D. Siegel D. (2021). The diverse functionality of NQO1 and its roles in redox control. Redox Biol. 41, 101950. 10.1016/J.REDOX.2021.101950 33774477 PMC8027776

[B160] Roy R. G. Mandal P. K. Maroon J. C. (2023). Oxidative stress occurs prior to amyloid Aβ plaque formation and tau phosphorylation in alzheimer’s disease: role of glutathione and metal ions. ACS Chem. Neurosci. 14, 2944–2954. 10.1021/ACSCHEMNEURO.3C00486 37561556 PMC10485904

[B161] Saha S. Buttari B. Profumo E. Tucci P. Saso L. (2021). A perspective on Nrf2 signaling pathway for neuroinflammation: a potential therapeutic target in alzheimer’s and Parkinson’s diseases. Front. Cell Neurosci. 15, 787258. 10.3389/fncel.2021.787258 35126058 PMC8813964

[B162] Sarlette A. Krampfl K. Grothe C. Neuhoff N. V. Dengler R. Petri S. (2008). Nuclear erythroid 2-related factor 2-antioxidative response element signaling pathway in motor cortex and spinal cord in amyotrophic lateral sclerosis. J. Neuropathol. Exp. Neurol. 67, 1055–1062. 10.1097/NEN.0B013E31818B4906 18957896

[B163] Satoh T. Okamoto S. I. Cui J. Watanabe Y. Furuta K. Suzuki M. (2006). From the Cover: activation of the Keap1/Nrf2 pathway for neuroprotection by electrophillic phase II inducers. Proc. Natl. Acad. Sci. U. S. A. 103, 768–773. 10.1073/PNAS.0505723102 16407140 PMC1334635

[B164] Scarffe L. A. Stevens D. A. Dawson V. L. Dawson T. M. (2014). Parkin and PINK1: much more than mitophagy. Trends Neurosci. 37, 315–324. 10.1016/J.TINS.2014.03.004 24735649 PMC4075431

[B165] Schepici G. Bramanti P. Mazzon E. (2020). Efficacy of sulforaphane in neurodegenerative diseases. Int. J. Mol. Sci. 21, 8637. 10.3390/IJMS21228637 33207780 PMC7698208

[B166] Schmidlin C. J. Dodson M. B. Madhavan L. Zhang D. D. (2019). Redox regulation by NRF2 in aging and disease. Free Radic. Biol. Med. 134, 702–707. 10.1016/J.FREERADBIOMED.2019.01.016 30654017 PMC6588470

[B167] Schulze-Topphoff U. Varrin-Doyer M. Pekarek K. Spencer C. M. Shetty A. Sagan S. A. (2016). Dimethyl fumarate treatment induces adaptive and innate immune modulation independent of Nrf2. Proc. Natl. Acad. Sci. U. S. A. 113, 4777–4782. 10.1073/pnas.1603907113 27078105 PMC4855599

[B168] Scoles D. R. Minikel E. V. Pulst S. M. (2019). Antisense oligonucleotides: a primer. Neurol. Genet. 5, e323. 10.1212/NXG.0000000000000323 31119194 PMC6501637

[B169] Shahcheraghi S. H. Salemi F. Peirovi N. Ayatollahi J. Alam W. Khan H. (2022). Nrf2 regulation by curcumin: molecular aspects for therapeutic prospects. Molecules 27, 167. 10.3390/MOLECULES27010167 PMC8746993 35011412

[B170] Shi X. Xiang S. Cao J. Zhu H. Yang B. He Q. (2019). Kelch-like proteins: physiological functions and relationships with diseases. Pharmacol. Res. 148, 104404. 10.1016/J.PHRS.2019.104404 31442578

[B171] Siebert A. Desai V. Chandrasekaran K. Fiskum G. Jafri M. S. (2009). Nrf2 activators provide neuroprotection against 6-hydroxydopamine toxicity in rat organotypic nigrostriatal cocultures. J. Neurosci. Res. 87, 1659–1669. 10.1002/JNR.21975 19125416

[B172] Smith T. S. Bennett J. P. (1997). Mitochondrial toxins in models of neurodegenerative diseases. I: *in vivo* brain hydroxyl radical production during systemic MPTP treatment or following microdialysis infusion of methylpyridinium or azide ions. Brain Res. 765, 183–188. 10.1016/S0006-8993(97)00429-0 9313890

[B215] Sobue A. Komine O. Yamanaka K. (2023). Neuroinflammation in Alzheimer’s disease: microglial signature and their relevance to disease. Inflamm. Regen. 43, 26. 10.1186/s41232-023-00277-3 37165437 PMC10170691

[B173] Subedi L. Cho K. Park Y. U. Choi H. J. Kim S. Y. (2019). Sulforaphane-enriched broccoli sprouts pretreated by pulsed electric fields reduces neuroinflammation and ameliorates scopolamine-induced amnesia in mouse brain through its antioxidant ability via nrf2-HO-1 activation. Oxid. Med. Cell Longev. 2019, 3549274. 10.1155/2019/3549274 31049133 PMC6458888

[B216] Sun X. Suo X. Xia X. Yu C. Dou Y. (2022). Dimethyl Fumarate is a Potential Therapeutic Option for Alzheimer’s Disease. J. Alzheimer’s Dis. 85, 443–456. 10.3233/JAD-215074 34842188

[B174] Suzen S. Tucci P. Profumo E. Buttari B. Saso L. (2022). A pivotal role of Nrf2 in neurodegenerative disorders: a new way for therapeutic strategies. Pharmaceuticals 15, 692. 10.3390/PH15060692 35745610 PMC9227112

[B175] Tafuri F. Ronchi D. Magri F. Comi G. P. Corti S. (2015). SOD1 misplacing and mitochondrial dysfunction in amyotrophic lateral sclerosis pathogenesis. Front. Cell Neurosci. 9, 336. 10.3389/fncel.2015.00336 26379505 PMC4548205

[B176] Tak Y. J. Park J. H. Rhim H. Kang S. (2020). ALS-related mutant SOD1 aggregates interfere with mitophagy by sequestering the autophagy receptor optineurin. Int. J. Mol. Sci. 21, 7525. 10.3390/IJMS21207525 33065963 PMC7590160

[B177] Tarozzi A. Morroni F. Merlicco A. Hrelia S. Angeloni C. Cantelli-Forti G. (2009). Sulforaphane as an inducer of glutathione prevents oxidative stress-induced cell death in a dopaminergic-like neuroblastoma cell line. J. Neurochem. 111, 1161–1171. 10.1111/J.1471-4159.2009.06394.X 19780897

[B178] Taylor J. P. Brown R. H. Cleveland D. W. (2016). Decoding ALS: from genes to mechanism. Nature 539 (7628), 197–206. 10.1038/nature20413 27830784 PMC5585017

[B179] Tortelli R. Zecca C. Piccininni M. Benmahamed S. Dell’Abate M. T. Barulli M. R. (2020). Plasma inflammatory cytokines are elevated in ALS. Front. Neurol. 11, 552295. 10.3389/fneur.2020.552295 33281700 PMC7691268

[B180] Townsend B. E. Johnson R. W. (2016). Sulforaphane induces Nrf2 target genes and attenuates inflammatory gene expression in microglia from brain of young adult and aged mice. Exp. Gerontol. 73, 42–48. 10.1016/J.EXGER.2015.11.004 26571201 PMC4713291

[B181] Tresse E. Marturia-Navarro J. Sew W. Q. G. Cisquella-Serra M. Jaberi E. Riera-Ponsati L. (2023). Mitochondrial DNA damage triggers spread of Parkinson’s disease-like pathology. Mol. Psychiatry 2023, 4902–4914. 10.1038/s41380-023-02251-4 PMC10914608 37779111

[B182] Tresse E. Riera-Ponsati L. Jaberi E. Sew W. Q. G. Ruscher K. Issazadeh-Navikas S. (2021). IFN-β rescues neurodegeneration by regulating mitochondrial fission via STAT5, PGAM5, and Drp1. EMBO J. 40, e106868. 10.15252/embj.2020106868 33913175 PMC8167366

[B183] Uruno A. Matsumaru D. Ryoke R. Saito R. Kadoguchi S. Saigusa D. (2020). Nrf2 suppresses oxidative stress and inflammation in app knock-in alzheimer’s disease model mice. Mol. Cell Biol. 40, e00467. 10.1128/MCB.00467-19 31932477 PMC7048263

[B184] Valente E. M. Abou-Sleiman P. M. Caputo V. Muqit M. M. K. Harvey K. Gispert S. (2004). Hereditary early-onset Parkinson’s disease caused by mutations in PINK1. Science 304, 1158–1160. 10.1126/science.1096284 15087508

[B185] Vargas M. R. Burton N. C. Gan L. Johnson D. A. Schäfer M. Werner S. (2013). Absence of Nrf2 or its selective overexpression in neurons and muscle does not affect survival in ALS-linked mutant hSOD1 mouse models. PLoS One 8, e56625. 10.1371/JOURNAL.PONE.0056625 23418589 PMC3572065

[B186] Vargas M. R. Johnson D. A. Sirkis D. W. Messing A. Johnson J. A. (2008). Nrf2 activation in astrocytes protects against neurodegeneration in mouse models of familial amyotrophic lateral sclerosis. J. Neurosci. 28, 13574–13581. 10.1523/JNEUROSCI.4099-08.2008 19074031 PMC2866507

[B187] Vauzour D. Buonfiglio M. Corona G. Chirafisi J. Vafeiadou K. Angeloni C. (2010). Sulforaphane protects cortical neurons against 5-S-cysteinyl-dopamine-induced toxicity through the activation of ERK1/2, Nrf-2 and the upregulation of detoxification enzymes. Mol. Nutr. Food Res. 54, 532–542. 10.1002/MNFR.200900197 20166144

[B188] Wang L. He C. (2022). Nrf2-mediated anti-inflammatory polarization of macrophages as therapeutic targets for osteoarthritis. Front. Immunol. 13, 967193. 10.3389/FIMMU.2022.967193 36032081 PMC9411667

[B189] Wang L. Li B. Quan M. Y. Li L. Chen Y. Tan G. J. (2017). Mechanism of oxidative stress p38MAPK-SGK1 signaling axis in experimental autoimmune encephalomyelitis (EAE). Oncotarget 8, 42808–42816. 10.18632/ONCOTARGET.17057 28467798 PMC5522107

[B190] Wang W. Wei C. Quan M. Li T. Jia J. (2020). Sulforaphane reverses the amyloid-β oligomers induced depressive-like behavior. J. Alzheimer’s Dis. 78, 127–137. 10.3233/JAD-200397 32925042

[B191] WHO (2023). Dementia. Available at: https://www.who.int/news-room/fact-sheets/detail/dementia (Accessed March 20, 2024).

[B192] Wierinckx A. Brevé J. Mercier D. Schultzberg M. Drukarch B. Van Dam A. M. (2005). Detoxication enzyme inducers modify cytokine production in rat mixed glial cells. J. Neuroimmunol. 166, 132–143. 10.1016/J.JNEUROIM.2005.05.013 15993952

[B193] Williamson T. P. Johnson D. A. Johnson J. A. (2012). Activation of the Nrf2-ARE pathway by siRNA knockdown of Keap1 reduces oxidative stress and provides partial protection from MPTP-mediated neurotoxicity. Neurotoxicology 33, 272–279. 10.1016/J.NEURO.2012.01.015 22342405 PMC3521526

[B194] Wilson D. M. Cookson M. R. Van Den Bosch L. Zetterberg H. Holtzman D. M. Dewachter I. (2023). Hallmarks of neurodegenerative diseases. Cell 186, 693–714. 10.1016/j.cell.2022.12.032 36803602

[B195] Witte M. E. Nijland P. G. Drexhage J. A. R. Gerritsen W. Geerts D. Van Het Hof B. (2013). Reduced expression of PGC-1α partly underlies mitochondrial changes and correlates with neuronal loss in multiple sclerosis cortex. Acta Neuropathol. 125, 231–243. 10.1007/s00401-012-1052-y 23073717

[B196] Wood-Allum C. A. Barber S. C. Kirby J. Heath P. Holden H. Mead R. (2006). Impairment of mitochondrial anti-oxidant defence in SOD1-related motor neuron injury and amelioration by ebselen. Brain 129, 1693–1709. 10.1093/BRAIN/AWL118 16702190

[B197] Yadav E. Yadav P. Khan M. M. U. Singh H. O. Verma A. (2022). Resveratrol: a potential therapeutic natural polyphenol for neurodegenerative diseases associated with mitochondrial dysfunction. Front. Pharmacol. 13, 922232. 10.3389/FPHAR.2022.922232 36188541 PMC9523540

[B198] Yagishita Y. Fahey J. W. Dinkova-Kostova A. T. Kensler T. W. (2019). Broccoli or sulforaphane: is it the source or dose that matters? Molecules 24, 3593. 10.3390/MOLECULES24193593 31590459 PMC6804255

[B199] Yang X. X. Yang R. Zhang F. (2022). Role of Nrf2 in Parkinson’s disease: toward new perspectives. Front. Pharmacol. 13, 919233. 10.3389/FPHAR.2022.919233 35814229 PMC9263373

[B200] Yang Z. B. Chen W. W. Chen H. P. Cai S. X. Lin J. D. Qiu L. Z. (2018). MiR-155 aggravated septic liver injury by oxidative stress-mediated ER stress and mitochondrial dysfunction via targeting Nrf-2. Exp. Mol. Pathol. 105, 387–394. 10.1016/J.YEXMP.2018.09.003 30218645

[B201] Zamiri K. Kesari S. Paul K. Hwang S. H. Hammock B. Kaczor-Urbanowicz K. E. (2023). Therapy of autoimmune inflammation in sporadic amyotrophic lateral sclerosis: dimethyl fumarate and H-151 downregulate inflammatory cytokines in the cGAS-STING pathway. FASEB J. 37, e23068. 10.1096/FJ.202300573R 37436778 PMC10619685

[B202] Zhang D. D. Hannink M. (2003). Distinct cysteine residues in Keap1 are required for Keap1-dependent ubiquitination of Nrf2 and for stabilization of Nrf2 by chemopreventive agents and oxidative stress. Mol. Cell Biol. 23, 8137–8151. 10.1128/MCB.23.22.8137-8151.2003 14585973 PMC262403

[B203] Zhang H. Thorwald M. A. D’agostino C. Morgan T. E. Finch C. E. Forman H. J. (2021). Inhibiting Bach1 enhanced the activation of Nrf2 signaling and the degradation of HNE in response to oxidative stress. Alzheimer’s Dementia 17, e053235. 10.1002/ALZ.053235

[B204] Zhang H. Zhou L. Davies K. J. A. Forman H. J. (2019). Silencing Bach1 alters aging-related changes in the expression of Nrf2-regulated genes in primary human bronchial epithelial cells. Arch. Biochem. Biophys. 672, 108074. 10.1016/J.ABB.2019.108074 31422075

[B205] Zhang R. Miao Q. W. Zhu C. X. Zhao Y. Liu L. Yang J. (2015). Sulforaphane ameliorates neurobehavioral deficits and protects the brain from amyloid β deposits and peroxidation in mice with Alzheimer-like lesions. Am. J. Alzheimers Dis. Other Demen 30, 183–191. 10.1177/1533317514542645 25024455 PMC10852928

[B206] Zhang S. Y. Gui L. N. Liu Y. Y. Shi S. Cheng Y. (2020). Oxidative stress marker aberrations in multiple sclerosis: a meta-analysis study. Front. Neurosci. 14, 823. 10.3389/fnins.2020.00823 32982663 PMC7479227

[B207] Zhang Y. Talalay P. Cho C. G. Posner G. H. (1992). A major inducer of anticarcinogenic protective enzymes from broccoli: isolation and elucidation of structure. Proc. Natl. Acad. Sci. U. S. A. 89, 2399–2403. 10.1073/PNAS.89.6.2399 1549603 PMC48665

[B208] Zhao F. Zhang J. Chang N. (2018). Epigenetic modification of Nrf2 by sulforaphane increases the antioxidative and anti-inflammatory capacity in a cellular model of Alzheimer’s disease. Eur. J. Pharmacol. 824, 1–10. 10.1016/J.EJPHAR.2018.01.046 29382536

[B209] Zheng W. Li X. Zhang T. Wang J. (2022). Biological mechanisms and clinical efficacy of sulforaphane for mental disorders. Gen. Psychiatr. 35, e100700. 10.1136/GPSYCH-2021-100700 35492261 PMC8987744

[B210] Zhou L. Zhang H. Davies K. J. A. Forman H. J. (2018). Aging-related decline in the induction of Nrf2-regulated antioxidant genes in human bronchial epithelial cells. Redox Biol. 14, 35–40. 10.1016/J.REDOX.2017.08.014 28863281 PMC5576992

[B211] Zhou Q. Chen B. Wang X. Wu L. Yang Y. Cheng X. (2016). Sulforaphane protects against rotenone-induced neurotoxicity *in vivo*: involvement of the mTOR, Nrf2, and autophagy pathways. Sci. Rep. 6, 32206. 10.1038/SREP32206 27553905 PMC4995453

[B212] Zhuang C. Wu Z. Xing C. Miao Z. (2017). Small molecules inhibiting Keap1–Nrf2 protein–protein interactions: a novel approach to activate Nrf2 function. Medchemcomm 8, 286–294. 10.1039/C6MD00500D 30108745 PMC6072482

